# CDK8 and CDK19 regulate intestinal differentiation and homeostasis via the chromatin remodeling complex SWI/SNF

**DOI:** 10.1172/JCI158593

**Published:** 2022-10-17

**Authors:** Marius V. Dannappel, Danxi Zhu, Xin Sun, Hui Kheng Chua, Marle Poppelaars, Monica Suehiro, Subash Khadka, Terry C.C. Lim Kam Sian, Dhanya Sooraj, Melissa Loi, Hugh Gao, Daniel Croagh, Roger J. Daly, Pouya Faridi, Thomas G. Boyer, Ron Firestein

**Affiliations:** 1 Centre for Cancer Research, Hudson Institute of Medical Research, Clayton, Victoria, Australia.; 2 Department of Molecular and Translational Science and; 3 Molecular and Translational Science, Faculty of Medicine, Nursing and Health Sciences, Monash University, Clayton, Victoria, Australia.; 4 Department of Molecular Medicine, Institute of Biotechnology, University of Texas Health Science Center at San Antonio, San Antonio, Texas, USA.; 5 Cancer Program, Biomedicine Discovery Institute and Department of Biochemistry and Molecular Biology,; 6 School of Clinical Sciences at Monash Health, and; 7 Department of Medicine, School of Clinical Sciences at Monash Health, Faculty of Medicine, Nursing and Health Sciences, Monash University, Clayton, Victoria, Australia.

**Keywords:** Gastroenterology, Genetics, Molecular genetics, Mouse models, Oncogenes

## Abstract

Initiation and maintenance of transcriptional states are critical for controlling normal tissue homeostasis and differentiation. The cyclin dependent kinases CDK8 and CDK19 (Mediator kinases) are regulatory components of Mediator, a highly conserved complex that orchestrates enhancer-mediated transcriptional output. While Mediator kinases have been implicated in the transcription of genes necessary for development and growth, its function in mammals has not been well defined. Using genetically defined models and pharmacological inhibitors, we showed that CDK8 and CDK19 function in a redundant manner to regulate intestinal lineage specification in humans and mice. The Mediator kinase module bound and phosphorylated key components of the chromatin remodeling complex switch/sucrose non-fermentable (SWI/SNF) in intestinal epithelial cells. Concomitantly, SWI/SNF and MED12-Mediator colocalized at distinct lineage-specifying enhancers in a CDK8/19–dependent manner. Thus, these studies reveal a transcriptional mechanism of intestinal cell specification, coordinated by the interaction between the chromatin remodeling complex SWI/SNF and Mediator kinase.

## Graphical abstract

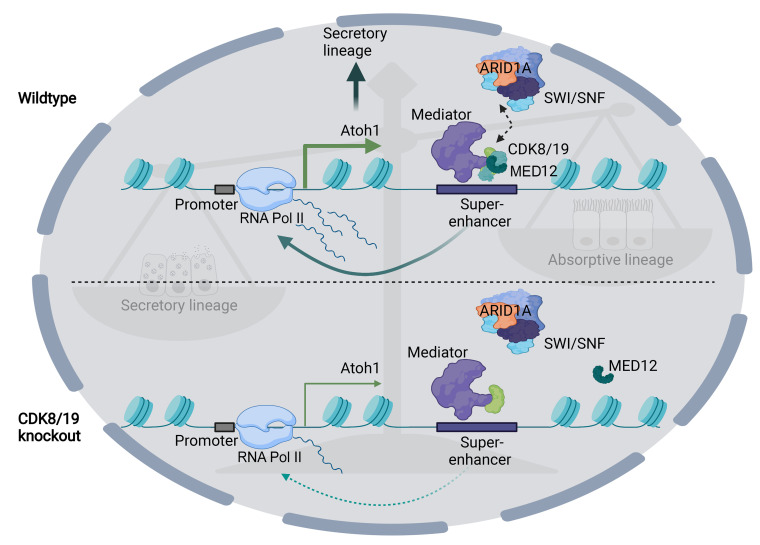

## Introduction

The intestinal epithelium is among the most proliferative tissues, with a complete turnover of intestinal epithelial cells (IECs) every 3 to 5 days. The self-renewal capacity of the intestinal epithelium relies on intestinal stem cells (ISCs), which reside at the bottom of specialized invaginations called crypts of Lieberkühn (1). ISCs continuously divide and give rise to transient amplifying cells while migrating upward through the villus toward the lumen and differentiating into mature lineages such as absorptive enterocytes as well as secretory goblet cells, tuft cells, and enteroendocrine cells (1). One exception to this migration pattern are Paneth cells, which escape the upward migration and instead move down to the bottom of the crypts, where they intersperse with ISCs to support their proliferative capacity (1, 2). ISC proliferation, migration of IECs along the crypt-villus axis, and their differentiation are controlled by the coordinated activity of morphogen-driven pathways such as BMP, EGF, Notch, and Wnt/β-catenin signaling. Notch signaling is the main pathway determining secretory cell differentiation. Cells with active Notch signaling express the transcription factor hairy and enhancer of split-1 (HES1), resulting in atonal BHLH transcription factor 1 (ATOH1) repression and commitment toward absorptive lineages, while cells that escape Notch signaling express ATOH1 and differentiate into the secretory lineages.

Higher-order chromatin structure and organization play an important role in differentiation and cell fate decisions in embryonic stem cells and in adult stem cells during tissue homeostasis (3–5). Accessible *cis*-regulatory elements provide binding sites for transcription factors (TFs) (6) to induce lineage-restricted gene expression programs that enable differentiation of tissue-specific cell lineages (7–9). Intestinal secretory and absorptive progenitors were shown to have a similar degree of accessible and permissive chromatin at the majority of *cis*-regulatory elements, suggesting that chromatin accessibility at *cis* elements may set the stage, but TFs such as ATOH1 ultimately control lineage specification (10). In addition, chromatin modifying complexes such as switch/sucrose non-fermentable (SWI/SNF), NuRD, and SRCAP have been linked to intestinal differentiation networks (11–15), underscoring the importance of chromatin structural dynamics in this process. Nevertheless, the detailed mechanisms determining chromatin access, and TF activity accounting for the high degree of intestinal cell lineage plasticity, remain largely unknown.

The multimeric Mediator complex is implicated in a number of steps of gene transcription, ranging from chromatin organization to RNA polymerase II phosphorylation (16). The Mediator kinase module composed of cyclin-dependent kinase 8 (CDK8), cyclin C (CCNC), Mediator complex subunit 12 (MED12), and MED13, reversibly associates with the core Mediator complex and functions as a catalytic subunit to activate or repress transcription by regulating TF activity via phosphorylation and colocalizing genome-wide at promoters and super-enhancers (17). Precise regulation and different subunit interactions are required for appropriate control of Mediator kinase activity. Consequently, mutation or changes in subunit expression are associated with aberrant activity of the kinase module, resulting in oncogenic signaling fueling tumor growth (18). For example, *CDK8* was identified as a colorectal cancer (CRC) oncogene with copy number gains driving tumor growth by positively regulating β-catenin target gene expression (19). Thus, the possibility of inhibiting CDK8/19 to treat CRC has gained traction in the last few years, and several Mediator kinase inhibitors have been developed.

Vertebrates express a paralog of CDK8, called CDK19, which shares 91% overall sequence homology to CDK8 with a nearly identical cyclin binding domain and kinase domain (20). CDK19 was shown to form a separate kinase module in a mutually exclusive manner with respect to the CDK8 kinase module (21). Consequently, CDK8 and CDK19 were shown to have clearly distinct functions as transcriptional regulators in different stimulus-specific pathways by directly interacting with TFs and activating distinct gene sets within the same transcriptional network (22–27).

Deletion of CDK8 alone, or inhibition of its kinase activity, is compatible with cell viability in cell lines (22, 28). Likewise, both intestine-specific and whole-body deletion of CDK8 in adult mice was well tolerated (29). Studies using a CDK8/19 inhibitor revealed sporadic body weight loss in mice and multiorgan toxicity in rats and dogs (30). Follow-up studies suggest that this inhibitor toxicity may be due to off-target effects (31), underscoring the urgent need to better define the function of the Mediator kinases in vivo.

Herein, we generated a series of genetically engineered, inducible mouse models enabling us to test the consequences of CDK8/19 deletion or kinase activity loss in the intestinal epithelium. We find that CDK8/19 deletion in the intestinal epithelium led to severe depletion of the secretory lineage compartment in both animal and ex vivo organoid models. While mice tolerated ablation of Mediator kinases in the intestine, intestinal organoid viability was severely affected owing to loss of Paneth cells. Using temporal RNA-Seq analyses and genome-wide ChIP-Seq studies in CDK8/CDK19-depleted IECs, we identify Mediator kinases as critical regulators of intestinal lineage differentiation. Phospho-proteomic analyses of the Mediator kinase complex targets converged to show a functional association between MED12-Mediator and SWI/SNF complexes that drives transcription from intestinal lineage–defined enhancers. In conclusion, these data highlight a role for Mediator kinases in regulating the intestinal secretory lineage, and demonstrate higher-order mechanisms of lineage differentiation in the intestine.

## Results

### CDK8 and CDK19 are dispensable for basal intestinal homeostasis.

We have previously reported that individual knockout of CDK8 is well tolerated in the intestinal epithelium (29). We hypothesized that the paralog gene, *CDK19*, may compensate for CDK8 loss. To begin to address this possibility, we first performed in silico analyses of publicly available scRNA-Seq data sets delineating the intestinal cell types of normal mouse (32) and human intestinal epithelium (33). Indeed, *CDK8* and *CDK19* were found to be coexpressed in nearly all cell lineages, albeit *CDK8* was generally more highly expressed than *CDK19* (Supplemental Figure 1, A–D; supplemental material available online with this article; https://doi.org/10.1172/JCI158593DS1). This raised the intriguing possibility that the Mediator kinases, CDK8 and CDK19, based on their high degree of sequence homology (20) and coexpression, may have functional redundancy in the intestinal epithelium.

**Figure 1:**
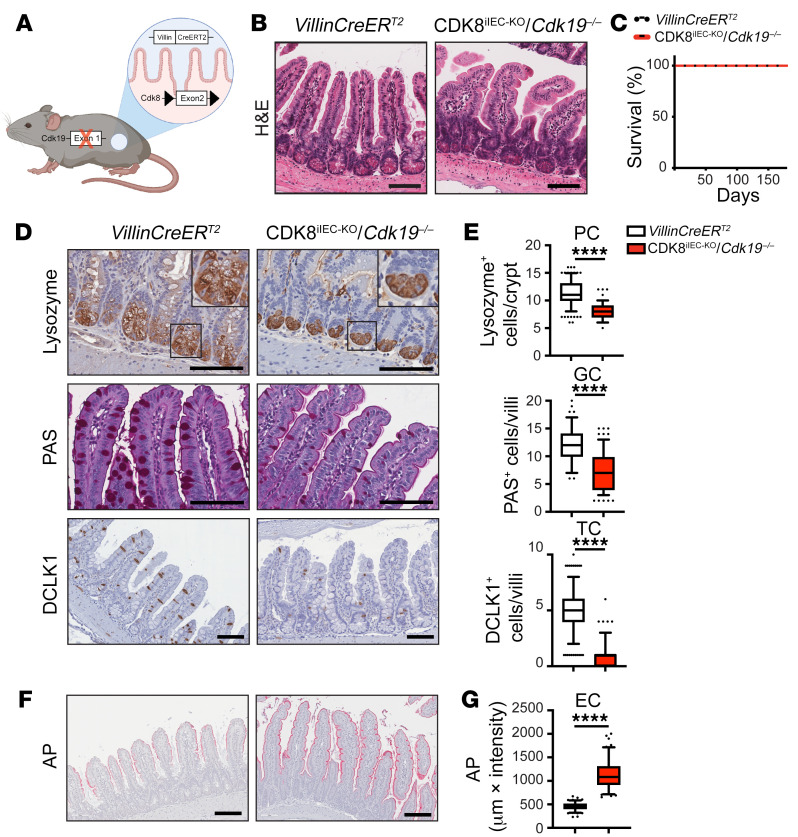
Deletion of CDK8/19 leads to defects in intestinal differentiation. (**A**) Schematic depicting the genetic mouse model used in this study. (**B**) H&E-stained ileal sections from mice with the indicated genotypes 3 weeks after tamoxifen injections (*VillinCreER^T2^*, *n =* 4; CDK8^iIEC-KO^/*Cdk19^–/–^*, *n =* 4). Scale bars: 100 μm. (**C**) Kaplan-Meier survival curve for tamoxifen-injected *VillinCreER^T2^* (*n =* 5) and CDK8^iIEC-KO^/*Cdk19^–/–^* mice (*n =* 5). Time after injection is indicated. (**D**) IHC for lysozyme, DCLK1, and periodic acid–Schiff (PAS) staining on ileal sections from *VillinCreER^T2^* (*n =* 4) and CDK8^iIEC-KO^/*Cdk19^–/–^* mice (*n =* 4) 3 weeks after tamoxifen injections. Rectangles show magnified inserts. Scale bars: 100 μm. (**E**) Quantification of images from **D**. Lysozyme IHC for *VillinCreER^T2^* (*n =* 4 mice, *n =* 200 crypts) and CDK8^iIEC-KO^/*Cdk19^–/–^* mice (*n =* 4 mice, *n =* 114 crypts); PAS staining for *VillinCreER^T2^* (*n =* 4 mice, *n =* 152 villi) and CDK8^iIEC-KO^/*Cdk19^–/–^* mice (*n =* 4 mice, *n =* 190 villi); and DCLK1 IHC for *VillinCreER^T2^* (*n =* 4 mice, *n =* 375 villi) and CDK8^iIEC-KO^/*Cdk19^–/–^* mice (*n =* 4 mice, *n =* 430 villi). Mann-Whitney test (Lyz, PAS, DCLK1). PC, Paneth cells; GC, goblet cells; TC, tuft cells. (**F**) Alkaline phosphatase (AP) staining on ileal sections from *VillinCreER^T2^* (*n =* 4) and CDK8^iIEC-KO^/*Cdk19^–/–^* mice (*n =* 4) 3 weeks after tamoxifen injections. Rectangles show magnified inserts. Scale bars: 100 μm. (**G**) Quantification of AP staining for *VillinCreER^T2^* (*n =* 4 mice, *n =* 152 villi) and CDK8^iIEC-KO^/*Cdk19^–/–^* mice (*n =* 4 mice, *n =* 190 villi). Unpaired 2-tailed *t* test. EC, enterocyte cells. For box-and-whisker plots, boxes depict 25th–75th percentile, whiskers show 5th–95th percentile, and points show outliers. ****P ≤ 0.001.

To assess the phenotypic and molecular consequence of CDK8/19 loss in the intestine, we generated a suite of genetically engineered mouse models harboring knockout and kinase-dead knockin alleles of CDK8 and CDK19. Similarly to CDK8-knockout mice (29), we found that *Cdk19^–/–^* mice (procured from The Jackson Laboratory) are viable and generally healthy (Supplemental Figure 2A). We then generated mice with compound deletion of Mediator kinases CDK8 and CDK19 in the intestinal epithelium by crossing *Cdk19^–/–^* mice, *Cdk8^fl/fl^* mice (29), and *VillinCreER^T2^* mice (34). The resulting *VillinCreER^T2^*/*Cdk8^fl/fl^*/*Cdk19^–/–^* mouse model enables a tamoxifen-inducible deletion of CDK8 specifically in IECs (hereafter termed CDK8^iIEC-KO^/*Cdk19^–/–^* mice) (Figure 1A). Tamoxifen treatment resulted in complete ablation of CDK8 in IECs at the RNA and protein levels (Supplemental Figure 2, B–D). Macroscopically, CDK8^iIEC-KO^/*Cdk19^–/–^* mice were indistinguishable from *VillinCreER^T2^*, CDK8^iIEC-KO^, and *Cdk8^fl/fl^*/*Cdk19^–/–^* mice (Supplemental Figure 2E). H&E analysis of colonic and ileal sections showed no gross morphological changes between CDK8^iIEC-KO^/*Cdk19^–/–^*, CDK8^iIEC-KO^, *Cdk8^fl/fl^*/*Cdk19^–/–^*, and untreated *VillinCreER^T2^*/*Cdk8^fl/fl^*/*Cdk19^–/–^* mice at 9 days and 3 weeks after deletion (Figure 1B and Supplemental Figure 2, F and G; and data not shown). Mice lacking intestinal expression of Mediator kinases exhibited normal viability (Figure 1C) with no histopathological changes observed even 6 months after deletion (Supplemental Figure 2H). These results indicate that Mediator kinases are dispensable for steady-state intestinal viability.

### Intestinal loss of Mediator kinases leads to defective secretory lineage differentiation.

Mediator kinases have been intimately implicated in embryonic stem cell maintenance (24, 35). To investigate the role of CDK8/19 in intestinal differentiation, we delineate the stem cell, secretory, and absorptive cell lineages in the CDK8/CDK19-deficient intestine. IHC for lysozyme revealed reduced Paneth cell (PC) numbers in *Cdk8^IEC-KO^*/*Cdk19^–/–^* mice compared with *VillinCreER^T2^* mice (Figure 1, D and E). Likewise, CDK8^iIEC-KO^/*Cdk19^–/–^* mice exhibited significantly reduced numbers of goblet cells (periodic acid–Schiff stain) and tuft cells (doublecortin-like kinase 1 [DCLK1] IHC) (Figure 1, D and E, and Supplemental Figure 3, A and B). Conversely, absorptive cell marker staining for alkaline phosphatase (AP) was significantly increased in CDK8^iIEC-KO^/*Cdk19^–/–^* compared with *VillinCreER^T2^* mice (Figure 1, F and G). Olfactomedin 4–positive (OLFM4-positive) cells exhibited a patchy staining pattern in CDK8^iIEC-KO^/*Cdk19^–/–^* mice compared with *VillinCreER^T2^* mice (Supplemental Figure 3C). No difference in proliferation was observed in *VillinCreER^T2^* and *Cdk8^fl/fl^*/*Cdk19^–/–^* mice by Ki-67 staining (Supplemental Figure 3, D and E). These effects were temporally and spatially sustained in the intestinal tract of mice lacking CDK8/19, as CDK8^iIEC-KO^/*Cdk19^–/–^* mice exhibited reduced secretory compartment cell types in both the ileum (Supplemental Figure 3, F–O) and duodenum (Supplemental Figure 3, P and Q) 6 months after CDK8 deletion. Notably, *Cdk8^fl/fl^*/*Cdk19^–/–^* mice exhibited a mild (~12%) but statistically significant decrease in Lyz^+^ cells compared with *VillinCreER^T2^* mice at both 3-week and 6-month time points (Supplemental Figure 3, G and H). Together, these results demonstrate that Mediator kinases are necessary for intestinal secretory cell lineage differentiation, but that these effects may be compensated, under basal conditions, to maintain ISCs and basal intestinal viability.

### Mediator kinases are required for IEC-intrinsic growth.

**Figure 2:**
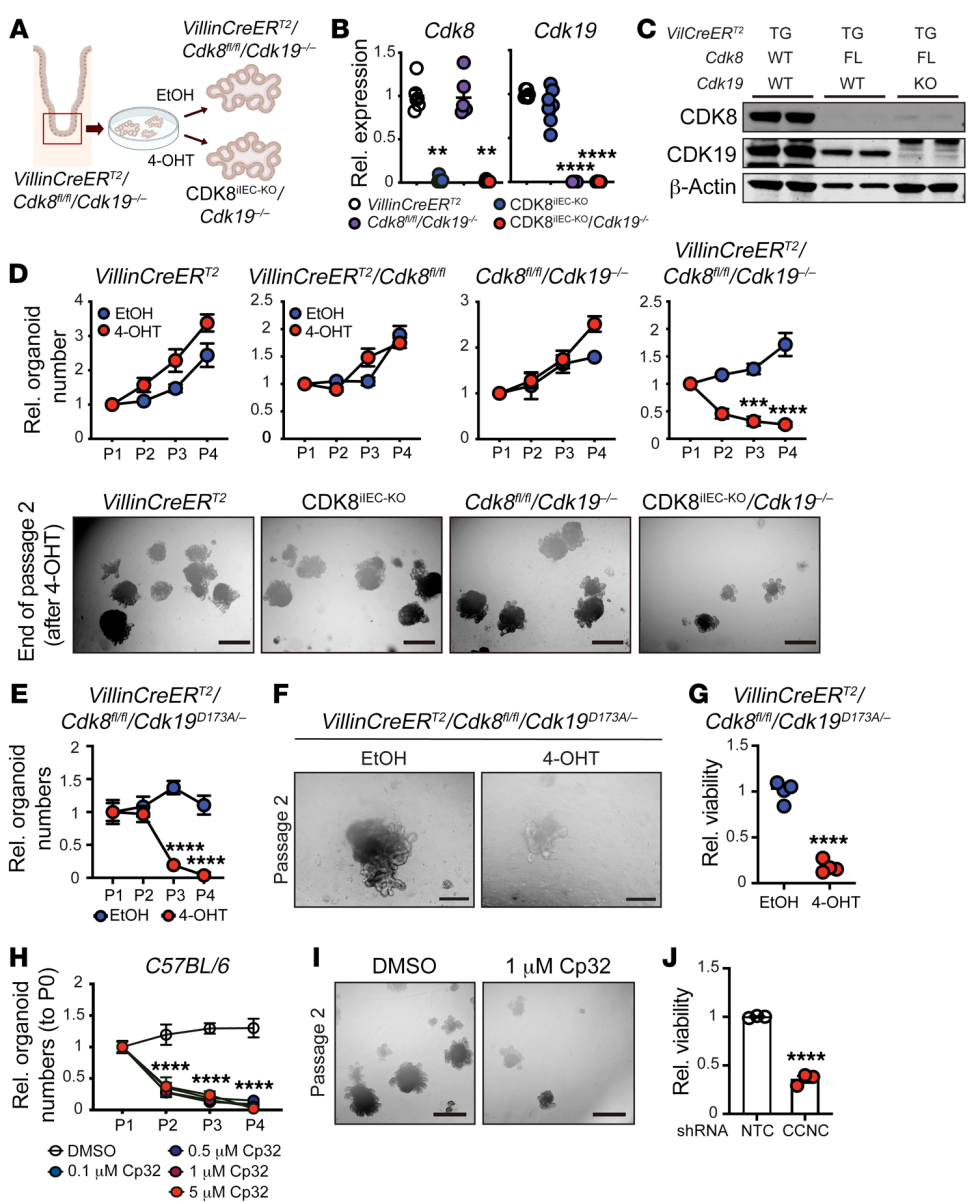
CDK8/19 kinase activity is necessary for intestinal epithelial cell intrinsic growth and differentiation. (**A**) Schematic representation of the genetic small intestinal organoid model. (**B**) qRT-PCR analysis of *Cdk8* and *Cdk19* gene expression from small intestinal organoids with the indicated genotypes 1 week after tamoxifen treatment. Representative data from 2 independent experiments. Holm-Šidák multiple-comparison test. (**C**) Immunoblot of small intestinal organoids from *VillinCreER^T2^*, *VillinCreER^T2^*/*Cdk8^fl/fl^*, and *VillinCreER^T2^*/*Cdk8^fl/fl^*/*Cdk19^–/–^* mice 1 week after 4-OHT treatment for 24 hours. (**D**) Top: Relative organoid numbers (to P1) of lines with the indicated genotypes for 4 passages after treatment with EtOH or 4-OHT. Bottom: Representative images of *VillinCreER^T2^*, CDK8^iIEC-KO^, *Cdk8^fl/fl^*/*Cdk19^–/–^*, and CDK8^iIEC-KO^/*Cdk19^–/–^* organoids at the end of passage 2 after 4-OHT treatment. Representative data from 3 independent experiments. Two-way ANOVA test (Šidák’s multiple-comparison test). Scale bars: 500 μm. (**E**) Relative numbers of EtOH- and 4-OHT–treated *VillinCreER^T2^*/*Cdk8^fl/fl^*/*Cdk19^D173A/–^* organoids for 4 passages. Representative data from 3 independent experiments. Unpaired 2-tailed *t* test. (**F**) Representative images of *VillinCreER^T2^*/*Cdk8^fl/fl^*/*Cdk19^D173A/–^* organoids at the end of passage 2 after treatment with EtOH or 4-OHT. Scale bars: 200 μm. (**G**) *VillinCreER^T2^*/*Cdk8^fl/fl^*/*Cdk19^D173A/–^* organoid viability at passage 2 after EtOH and 4-OHT treatment measured with alamarBlue HS (Thermo Fisher Scientific). Representative of 3 independent experiments. Unpaired 2-tailed *t* test. (**H**) Relative organoid numbers of intestinal organoids (C57BL/6 WT) treated with the indicated concentrations of Cp32 for 4 passages. Representative data from 3 independent experiments. Ordinary 1-way ANOVA. (**I**) Representative images of C57BL/6 small intestinal organoids treated with DMSO or 1 μM Cp32 at the end of passage 2. Scale bars: 500 μm. (**J**) Bar graph shows human small intestinal organoid viability (alamarBlue HS) 96 hours after transduction with shRNA targeting *CCNC* or nontargeting control. Unpaired 2-tailed *t* test. Data represent mean ± SEM. **P ≤ 0.01, ***P ≤ 0.005, ****P ≤ 0.001.

Previous reports have shown that the intestinal secretory lineage is dispensable for ISC maintenance and intestine proliferation in vivo, but not ex vivo, owing to compensatory Wnt signaling from the mesenchymal niche (36–38). To assess whether CDK8/19-deficient crypts sustain self-renewal and growth in the absence of the mesenchymal niche, we isolated small intestinal organoids from *VillinCreER^T2^*/*Cdk8^fl/fl^*/*Cdk19^–/–^* mice. We confirmed loss of CDK8 upon tamoxifen-induction in both *VillinCreER^T2^*/*Cdk8^fl/fl^*/*Cdk19^–/–^* and *VillinCreER^T2^*/*Cdk8^fl/fl^* organoids compared with *VillinCreER^T2^* controls (Figure 2, A–C, and Supplemental Figure 4, A and B). Strikingly, we found that loss of Mediator kinases led to a steady and profound reduction in both organoid number and size from 1 week after deletion with complete loss of organoid viability seen 4 weeks (passage 4) after the deletion of CDK8 (Figure 2D and Supplemental Figure 4, C–F). The results were confirmed in an additional 2 independently established organoid lines originating from different mice of the same genotype (Supplemental Figure 4, G and H). Importantly, these effects were specifically attributed to complete CDK8/19 loss, as neither single deletion of either CDK8 or CDK19, nor Cre activation alone, affected organoid growth (Figure 2D and Supplemental Figure 4, D–F). These results demonstrate functional redundancy for CDK8 and CDK19 in IEC-intrinsic growth and maintenance of the intestinal crypt.

CDK8/19 may act through both kinase-dependent and kinase-independent/scaffolding function (17). To test these possibilities in the context of intestinal differentiation, we generated and employed a knockin CDK19 kinase–dead mouse model (*Cdk19^D173A^*). As homozygous *Cdk19^D173A/D173A^* mice exhibited perinatal lethality (unpublished), heterozygous CDK19^D173A^ mice were used in which the second allele of CDK19 was constitutively deleted (*Cdk19^D173A/–^*) to generate *VillinCreER^T2^*/*Cdk8^fl/fl^*/*Cdk19^D173A/–^* mice. Importantly, the CDK19 kinase–dead allele was expressed at levels similar to those of WT CDK19 (Supplemental Figure 4I). Similarly to CDK8^iIEC-KO^/*Cdk19^–/–^* mice, CDK8^iIEC-KO^/*Cdk19^D173A/–^* mice did not exhibit any gross intestinal pathology (Supplemental Figure 4J). Small intestinal organoids were isolated from *VillinCreER^T2^*/*Cdk8^fl/fl^*/*Cdk19^D173A/–^* mice, and tamoxifen treatment resulted in efficient deletion of CDK8 (Supplemental Figure 4K). CDK8^iIEC-KO^/*Cdk19^D173A/–^* but not *VillinCreER^T2^*/*Cdk8^fl/fl^*/*Cdk19^D173A/WT^* organoids exhibited profoundly reduced organoid growth across several parametric measurements (i.e., number, size, viability) (Figure 2, E–G, and Supplemental Figure 4L). To confirm these effects, we used an orthogonal pharmacological approach. C57BL/6 organoids were treated with compound 32 (Cp32), a highly potent and specific CDK8/19 kinase inhibitor (39, 40). Consistent with our kinase-dead knockin genetic approach, WT organoids treated with different concentrations of CDK8/19 chemical inhibitor (Cp32) showed profoundly reduced viability (Figure 2, H and I) even at 0.1 μM. Cp32 on-target activity was confirmed by S727–phospho-STAT1 analysis using the lowest Cp32 concentration that showed viability effects (Supplemental Figure 4M). Importantly, these results were recapitulated in human small intestinal organoids where CDK8/19 was inhibited using orthogonal functional genomic and pharmacological inhibition. Organoids were isolated from duodenal biopsies from human patients and transduced with a lentiviral construct expressing a shRNA targeting CCNC, mimicking the effects of CDK8/19 kinase inhibition. Strikingly, CCNC depletion led to a significant reduction in intestinal organoid growth in comparison with organoids treated with nontargeting control (shNTC) (Figure 2J). CCNC depletion was validated by quantitative reverse transcriptase PCR (qRT-PCR) (Supplemental Figure 5A). Importantly, this growth defect was recapitulated when human intestinal organoids were treated with the CDK8/19 inhibitor, Cp32 (Supplemental Figure 5, B and C). Taken together, these complementary approaches identify that the kinase activity of CDK8/19 is essential for the maintenance of murine and human small intestinal organoids.

### Wnt3 is necessary and sufficient for restoring intestinal homeostasis in the absence of Mediator kinases.

**Figure 3:**
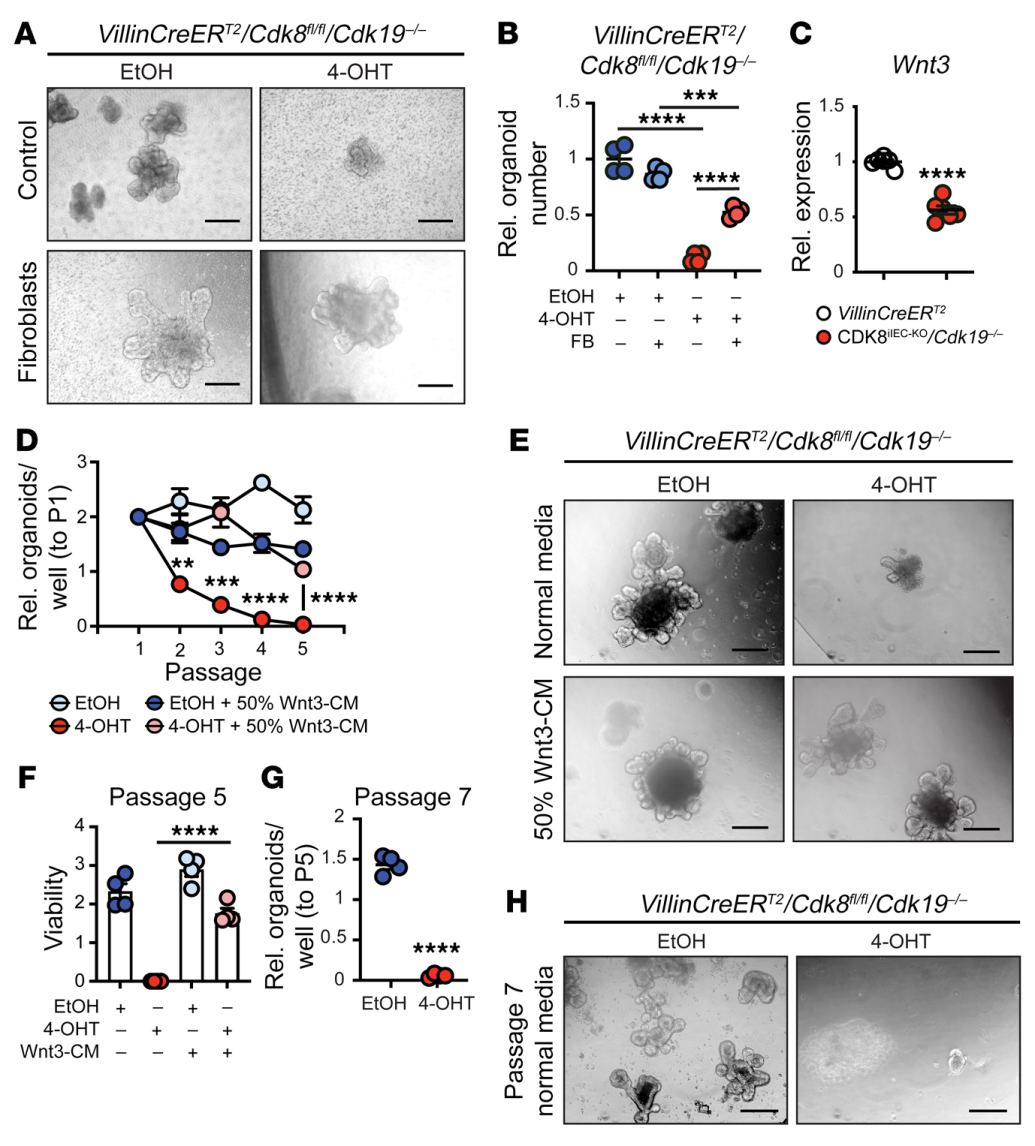
The Mediator kinase–dependent intestinal organoid growth defect is rescued by the intestinal niche and in a Wnt3-dependent manner. (**A** and **B**) Representative images (**A**) and relative organoid numbers (**B**) of EtOH- and 4-OHT–treated *VillinCreER^T2^*/*Cdk8^fl/fl^*/*Cdk19^–/–^* intestinal organoids cultured in the presence or absence of primary murine fibroblasts (FB). Ordinary 1-way ANOVA with Tukey’s multiple-comparison test. Scale bars: 200 μm. (**C**) qRT-PCR analysis of *Wnt3* expression in *VillinCreER^T2^* and *VillinCreER^T2^*/*Cdk8^fl/fl^*/*Cdk19^–/–^* organoids 1 week after 4-OHT treatment. Unpaired 2-tailed *t* test. Color-coded mouse genotypes are indicated below. (**D**) *VillinCreER^T2^*/*Cdk8^fl/fl^*/*Cdk19^–/–^* intestinal organoid numbers relative to passage 1 organoids treated with the indicated conditions. Unpaired 2-tailed *t* test. (**E** and **F**) Representative images (**E**) and viability (**F**) of *VillinCreER^T2^*/*Cdk8^fl/fl^*/*Cdk19^–/–^* organoids treated with EtOH or 4-OHT cultured in normal medium or 50% Wnt3-conditioned medium (CM) at the end of passage 2 after 4-OHT treatment. Holm-Šidák multiple-comparison test. Scale bars: 200 μm. (**G** and **H**) Proliferation (**G**) and representative images (**H**) of *VillinCreER^T2^*/*Cdk8^fl/fl^*/*Cdk19^–/–^* organoids at passage 7 after EtOH or 4-OHT and 2 passages after removal of Wnt3-CM. Unpaired 2-tailed *t* test. Scale bars: 200 μm. Data represent mean ± SEM. **P ≤ 0.01, ***P ≤ 0.005, ****P ≤ 0.001.

The intestinal mesenchyme is an important source of Wnt ligands that support ISCs and epithelial renewal (36–38). We hypothesized that the profound growth phenotype observed in CDK8/19–deficient intestinal crypts ex vivo compared with in vivo may be explained by an IEC-intrinsic function for CDK8/19 that can be compensated by the mesenchymal niche. To test this possibility, primary murine intestinal fibroblasts were cocultured together with Mediator kinase–deficient intestinal organoids to recapitulate extrinsic Wnt sources. Strikingly, we found that intestinal mesenchymal fibroblasts restored growth capacity of CDK8^iIEC-KO^/*Cdk19^–/–^* organoids (Figure 3, A and B). While PCs are dispensable for ISCs and intestinal homeostasis in vivo, they are essential for ISC maintenance in organoid cultures (2). Given the loss of PCs in CDK8/19–deficient intestines, we postulated that the growth defect observed in CDK8/19–deficient organoids may be caused by loss of PC-derived Wnt3. Consistently, qRT-PCR analysis showed significant downregulation of *Wnt3* in CDK8/19–deficient organoids 7 days after CDK8 deletion (Figure 3C).

To assess whether Wnt3 is sufficient to restore ISC maintenance and organoid growth, we cultured CDK8/19-deficient organoids in Wnt3-conditioned medium (Wnt3-CM) immediately after tamoxifen-induced CDK8 deletion. Indeed, exogenous Wnt3 rescued CDK8^iIEC-KO^/*Cdk19^–/–^* organoid growth and viability after CDK8 deletion (Figure 3, D–F). Lastly, survival of CDK8/19-deficient organoids depended on a continuous Wnt3 supply, as CDK8^iIEC-KO^/*Cdk19^–/–^* organoids collapsed within 2 passages of Wnt3 withdrawal (Figure 3, G and H). These data indicate that Wnt3 is both necessary and sufficient to rescue growth of CDK8/CDK19-deficient organoids and resolve the question of why CDK8^iIEC-KO^/*Cdk19^–/–^* mice do not show a visible defect in intestinal homeostasis under basal conditions.

### Mediator kinases regulate intestinal secretory transcriptional programs.

**Figure 4:**
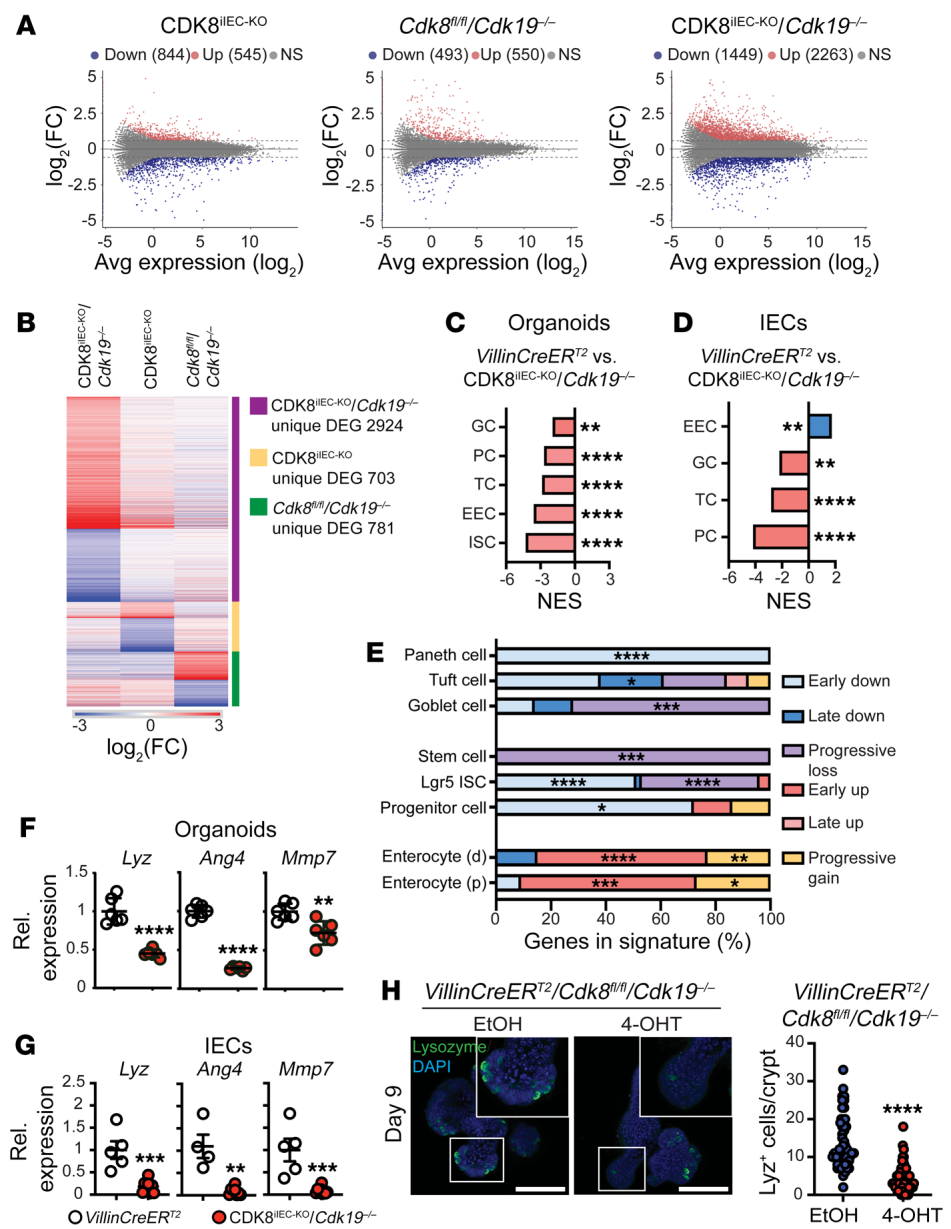
Deletion of CDK8/19 blocks secretory progenitor cell differentiation. (**A**) MA plots show differentially expressed genes in CDK8^iIEC-KO^ and CDK8^iIEC-KO^/*Cdk19^–/–^* intestinal organoids (vs. *VillinCreER^T2^*) and *Cdk8^fl/fl^*/*Cdk19^–/–^* organoids (vs. C57BL/6) 7 days after 4-OHT treatment. NS, not significant. (**B**) Heatmap depicts unique differentially expressed genes (DEGs) in organoids with the indicated genotypes 7 days after 4-OHT treatment. (**C**) GSEA for intestinal cell lineages shows normalized enrichment score (NES) comparing *VillinCreER^T2^* and CDK8^iIEC-KO^/*Cdk19^–/–^* organoids. GC, goblet cells; PC, Paneth cells; TC, tuft cells; EEC, enteroendocrine cells; ISC, intestinal stem cells. (**D**) GSEA for intestinal cell lineages shows NES comparing *VillinCreER^T2^* and CDK8^iIEC-KO^/*Cdk19^–/–^* small intestinal IECs. (**E**) Intestinal cell lineage signature and gene expression cluster analysis in 4-OHT–treated *VillinCreER^T2^*/*Cdk8^fl/fl^*/*Cdk19^–/–^* organoids compared with EtOH controls at days 3, 5, 7, and 10 after treatment. Fisher’s exact test with Bonferroni correction. (**F** and **G**) qRT-PCR analysis of the indicated Paneth cell marker expression in intestinal organoids 7 days after 4-OHT treatment (**F**) and small intestinal IECs from mice (**G**). Mouse genotypes are color-coded and indicated below **G**. Unpaired 2-tailed *t* test and Mann-Whitney test. (**H**) Immunofluorescent staining and quantification of lysozyme in *VillinCreER^T2^*/*Cdk8^fl/fl^*/*Cdk19^–/–^* organoids 9 days after EtOH and 4-OHT treatment. EtOH, *n =* 52 crypts; 4-OHT, *n =* 65 crypts. Scale bars: 50 μm. Mann-Whitney test. Data represent mean ± SEM. *P ≤ 0.05, **P ≤ 0.01, ***P ≤ 0.005, ****P ≤ 0.001.

Given the role of Mediator kinases in regulating RNA polymerase II–mediated transcription, we hypothesized that CDK8 and CDK19 directly act to regulate distinct transcriptional programs necessary for intestinal secretory cell differentiation. To determine the transcriptional output of Mediator kinase activity specifically in the intestine, we performed whole-transcriptome (RNA-Seq) analysis on intestinal organoids 7 days after individual or combined loss of CDK8/19. We found modest transcriptional changes in CDK8^iIEC-KO^ organoids (844 genes downregulated, 545 upregulated) and *Cdk8^fl/fl^*/*Cdk19^–/–^* organoids (493 genes downregulated, 550 upregulated). In contrast, CDK8^iIEC-KO^/*Cdk19^–/–^* organoids were marked by broader transcriptional perturbations with 3,712 differentially expressed genes (DEGs) (2,263 upregulated, 1,449 downregulated; fold change [FC] cutoff ±1.5, FDR < 0.05) (Figure 4A). Indeed, unsupervised clustering revealed that the vast majority of CDK8/19–regulated genes (2,924/3,712; 79%) were only found upon combined CDK8/19 deletion, highlighting their functional redundancy in intestinal transcriptional control (Figure 4B and Supplemental Figure 6, A and B). Lastly, we performed RNA-Seq on IECs derived from Mediator kinase–deficient (CDK8^iIEC-KO^/*Cdk19^–/–^*) and WT (*VillinCreER^T2^*) intestinal tissues 14 days after induced deletion and identified 970 DEGs (650 upregulated, 320 downregulated) (Supplemental Figure 6C and Supplemental Table 1).

Mediator kinases have been implicated in the regulation of Wnt signaling and stemness genes in CRC cells (19, 24). Indeed, gene expression analysis of 11 stem-related genes revealed that CDK8/19–depleted organoids showed reduced expression of the key stem cell and early progenitor markers *Olfm4*, *Ascl2*, and *Msi1* (Supplemental Figure 6D). Notably, *Olfm4* gene expression was also reduced in CDK8/19–deficient IECs (Supplemental Figure 6D), highlighting a potential role for CDK8/19 in *Olfm4* transcription. We then examined the effect of CDK8/19 loss on Wnt/β-catenin pathway–related gene expression using a signature of 93 Wnt pathway component genes. Strikingly, we found that only Wnt3 was downregulated significantly in organoids (log_2_FC < –1.0, FDR < 0.05) and trended down in IECs as well (log_2_FC < –1.0, FDR < 0.20) (Supplemental Figure 6E). Importantly, the Wnt3 downregulation in IECs was confirmed by qRT-PCR (Supplemental Figure 6F). This suggests that CDK8 and CDK19 do not play a direct role in regulating Wnt/β-catenin mediated transcription in the normal intestine.

Recent RNA-Seq studies mapping the intestine at the single-cell level (scRNA-Seq) have provided a transcriptomic atlas of cell state, helping to define cellular lineage and identity with unparalleled precision. We sought to delineate discrete cell types and clarify differentiation programs affected by loss of Mediator kinase. To do so, we performed gene set enrichment analysis (GSEA) on DEGs from CDK8/19–deficient organoids and IECs using cell type–defined gene sets from scRNA-Seq studies of mouse and human intestine (32, 33). GSEA showed a significant negative enrichment in multiple intestinal secretory lineage cell types and positive correlation with enterocyte signatures (Figure 4, C and D). These data are consistent with the skewed differentiation pattern observed in the intestines of CDK8^iIEC-KO^/*Cdk19^–/–^* mice and help define distinct cell states regulated by the activity of the Mediator kinases.

Intestinal differentiation is tightly regulated and defined by the acquisition of lineage-specific gene expression programs in a spatial and temporal manner (10, 41). To determine the transcriptional changes directly mediated by the Mediator kinases and infer causality, we performed a temporal transcriptomic analysis (RNA-Seq) on intestinal organoids at 3, 5, 7, and 10 days after ablation of Mediator kinases. DEGs were clustered based on 6 temporally defined expression patterns: downregulated early (log_2_FC < –1, days 3–5), downregulated late (log_2_FC < –1, days 5–7, days 7–10), upregulated early (log_2_FC > 1, days 3–5), upregulated late (log_2_FC > 1, days 5–7, days 7–10), stepwise/progressive loss (log_2_FC < –1, days 3–10), and stepwise/progressive gain (log_2_FC > 1, days 3–10) (Supplemental Figure 7A and Supplemental Table 2). GSEA of these 6 clusters with intestinal cell type signatures revealed distinct downregulation of progenitor and Paneth signatures as the earliest changes after loss of CDK8/19. Interestingly, loss of other intestinal secretory cell types (goblet cells, tuft cells) and stem cell signatures correlated with genes whose expression was more gradually lost (progressive loss signature) (Figure 4E). We confirmed these results using 3 well-established PC markers (*Lyz*, *Ang4*, *Mmp7*), which were highly downregulated in CDK8/19–deficient organoids and IECs (Figure 4, F and G, and Supplemental Figure 7B). Importantly, these results were specific to CDK8/19 depletion, as 4-hydroxytamoxifen (4-OHT) treatment of *VillinCreER^T2^* did not reduce secretory marker expression (Supplemental Figure 7C). Lastly, PC depletion was confirmed at the cellular level by Lyz immunofluorescence staining directly on CDK8^iIEC-KO^/*Cdk19^–/–^* organoids, confirming the transcriptomic data (Figure 4H).

To determine whether the CDK8/19 requirement for secretory cell types is Wnt3 dependent, we subjected RNA from EtOH- and 4-OHT–treated *VillinCreER^T2^*/*Cdk8^fl/fl^*/*Cdk19^–/–^* organoids cultured in Wnt3-CM for 10 days to whole-transcriptome sequencing (RNA-Seq). Gene expression signatures from CDK8/19–deficient organoids cultured in Wnt3-CM maintained a negative enrichment for PCs and other secretory cell lineages, but no longer showed negative enrichment for ISCs (Supplemental Figure 8A). The reduction in PC cells (Lyz, Ang4, Mmp7) and tuft cells (Pou2f3) was validated by qRT-PCR in CDK8/19–deficient organoids cocultured with fibroblasts and Wnt3-CM (Supplemental Figure 8, B–D, and Supplemental Table 1). These results suggest that CDK8/19 act independently of Wnt3 to produce the secretory cell types.

To assess whether these effects are kinase dependent, we characterized the secretory lineage in the Cdk19 kinase–dead–expressing mouse (*Cdk19^D173A^*). CDK8^iIEC-KO^/*Cdk19^D173A/–^* intestines showed a significantly lower number of PCs by IHC analysis (Supplemental Figure 8E) and reduced expression of the PC markers Lyz and Ang4 by qRT-PCR (Supplemental Figure 8F), phenocopying CDK8/19 protein depletion. To extend these findings, we performed whole-transcriptome (RNA-Seq) analysis on both CDK8^iIEC-KO^/*Cdk19^D173A/–^* mouse IECs and organoids. Strikingly, both IECs and organoids showed a significant negative enrichment of Paneth and tuft cell signatures (Supplemental Figure 8G). Lastly, we inhibited CDK8/19 activity using CCNC shRNA in human intestinal organoids and with Cp32 in both mouse and human intestinal cells. Consistent with our genetic model above, we observed loss of PC markers by qRT-PCR after both pharmacological (Cp32) and genetic (CCNC shRNA) kinase inhibition of CDK8/19 in mouse and human intestinal organoids (Supplemental Figure 8, H–J). Collectively, these data demonstrate an indispensable role for CDK8/19 kinase activity, specifically for PC production and more broadly for intestinal secretory lineage differentiation.

### Phospho-proteomic analysis reveals a functional interaction between the CDK8/19 kinase module and the SWI/SNF complex in the intestinal epithelium.

**Figure 5:**
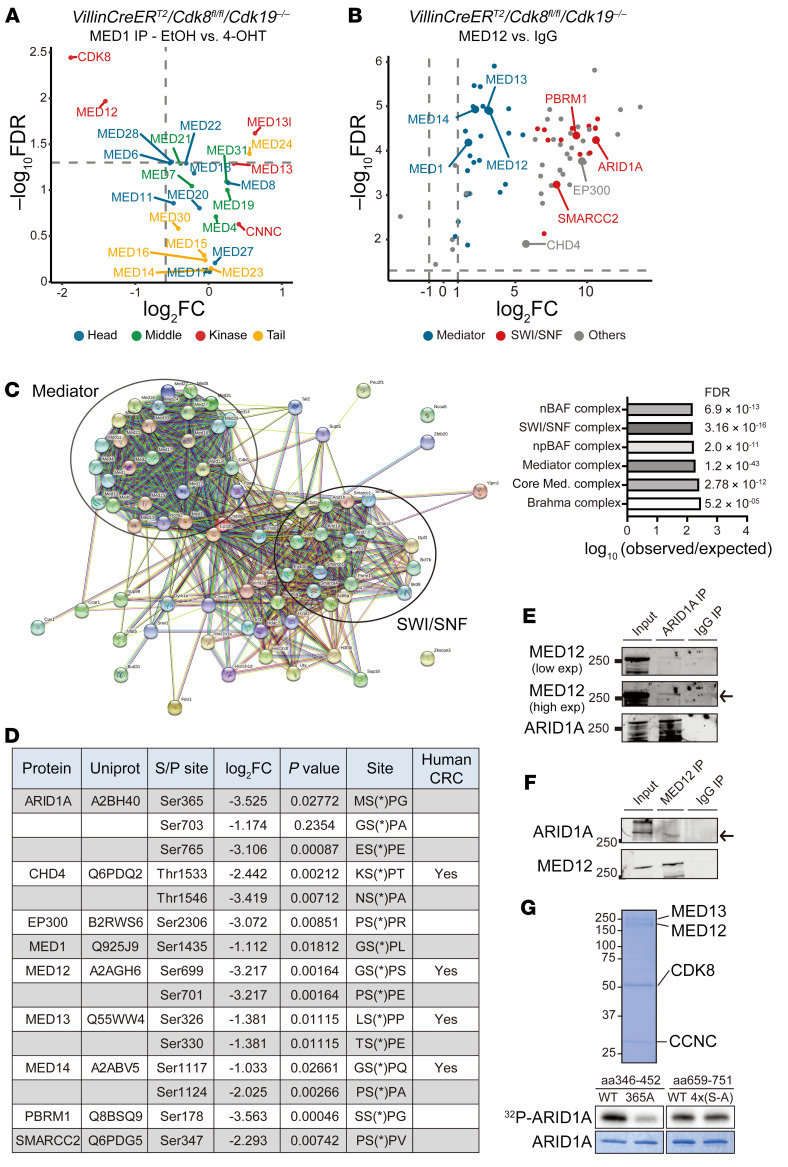
Phospho-proteomic analysis of MED12-Mediator identifies interactions with the SWI/SNF complex. (**A**) Quantitative proteomics (liquid chromatography–tandem mass spectrometry [LC-MS/MS]) after isobaric labeling of MED1-coimmunoprecipitated proteins in *VillinCreER^T2^*/*Cdk8^fl/fl^*/*Cdk19^–/–^* intestinal organoids 7 days after EtOH and 4-OHT treatment. Components of the Mediator complex are color-coded as indicated. (**B**) Quantitative proteomics (LC-MS/MS) after isobaric labeling of MED12-coimmunoprecipitated proteins in *VillinCreER^T2^*/*Cdk8^fl/fl^*/*Cdk19^–/–^* intestinal organoids. Mediator and SWI/SNF complex components are colored as indicated. (**C**) STRING analysis of MED12 interaction partners in EtOH-treated *VillinCreER^T2^*/*Cdk8^fl/fl^*/*Cdk19^–/–^* organoids and STRING cellular components (Gene Ontology) enrichment analysis. (**D**) Inset table shows identified phosphorylation sites reduced in 4-OHT–treated *VillinCreER^T2^*/*Cdk8^fl/fl^*/*Cdk19^–/–^* organoids compared with EtOH-treated controls 5 days after treatment (log_2_FC < –1, FDR < 0.05). (**E**) Coimmunoprecipitation (co-IP) assay in *VillinCreER^T2^* intestinal organoids. Immunoprecipitation antibody is ARID1A or IgG control; Western blot antibodies are MED12 and ARID1A. Arrow indicates coimmunoprecipitated protein. (**F**) Co-IP assay in *VillinCreER^T2^* intestinal organoids. Immunoprecipitation antibody is MED12 or IgG control; Western blot antibodies are MED12 and ARID1A. Arrow indicates coimmunoprecipitated protein. (**G**) Coomassie blue–stained gel shows reconstituted CDK8, MED12, and CCNC. Blot shows ^32^P-labeled protein in the presence of the kinase complex as indicated. 4x(S-A), ARID1A fragment (aa 659–751) containing S-to-A mutations on Ser696, Ser699, Ser703, and Ser716.

CDK8 and CDK19 have been shown to regulate transcription in both a Mediator-dependent and a Mediator-independent manner. To determine whether loss of Mediator kinases in the intestine leads to dysregulated core Mediator composition and activity, we performed quantitative (tandem mass tagging) immunoprecipitation-coupled mass spectrometry using Mediator components in WT and CDK8/19–deficient cells. Using an antibody directed at MED1, we coimmunoprecipitated the majority (22 of 26) of the core Mediator subunits at equivalent levels in both WT and CDK8/19–deficient intestinal organoids. Importantly, total levels of Mediator complex subunits were largely unaltered (Supplemental Figure 9A). Strikingly, however, CDK8/19 ablation led to dissolution of MED1/core Mediator from the kinase module protein MED12 (Figure 5A and Supplemental Table 3). These data suggest that loss of Mediator kinases perturbs the ability of MED12 to interact with core Mediator, without disrupting the composition of Mediator itself.

MED12 is a critical regulatory subunit of the Mediator kinase complex that is required for priming CDK8/19 kinase activity and linking CDK8/19 to additional kinase substrates (42, 43). Our work, herein, shows that the ability of Mediator kinases to control intestinal secretory cell differentiation is kinase dependent. To identify potential direct substrates of CDK8/19 activity required for intestinal differentiation, we took a 2-pronged approach. Firstly, we subjected WT and CDK8/19–depleted intestinal organoids to phospho-proteomic analysis. In total, 103 high-confidence phosphorylation events (in 68 unique nuclear proteins) were found to be significantly reduced upon loss of CDK8/19 (log_2_FC > 1, FDR < 0.05) (Supplemental Table 4). Of these 103 sites, 66 harbored canonical CDK active sites (SP/TP sites) (Supplemental Figure 9B), suggesting that they may be directly phosphorylated by CDK8/19. STRING network analysis of the 68 putative CDK8/19 kinase substrates revealed strong enrichment in chromatin remodeling and epigenetic regulatory complexes (Supplemental Figure 9, C and D).

To identify which of these proteins may be direct targets of Mediator kinases, we performed quantitative immunoprecipitation-coupled mass spectrometry in IECs using a MED12-directed antibody. After filtering for nuclear proteins, we found 72 significant MED12 binding partners (log_2_FC > 1, FDR < 0.05) compared with isotype IgG pull-down controls (Figure 5B and Supplemental Table 5). As expected, these included the majority of core Mediator and CDK8 kinase module components (23 of 28; 82%). Strikingly, we found that MED12 associated with several other enhancer regulatory proteins, most notably more than half of the SWI/SNF subunit proteins (Figure 5, B and C). Overlay of CDK8/19–regulated phospho-proteome and MED12 interactome analyses revealed 9 proteins, including 3 SWI/SNF subunits (ARID1A, SMARCC2, and PBRM1), that both bound to MED12 and were phosphorylated in the presence of Mediator kinases (Figure 5, B and D, and Supplemental Figure 9E). Importantly, 4 of the other 6 non–SWI/SNF subunits were also found as CDK8/19 targets in HCT116 CRC cells (44) (Figure 5D). Interestingly, HCT116 harbors mutations in SMARCA4 (BRG1), an important component of SWI/SNF complex function (45), providing a potential explanation of why these phosphorylation events were not identified in this cell line.

We confirmed MED12 and SWI/SNF complex interaction by performing reciprocal immunoprecipitation and Western blotting for MED12 and a key SWI/SNF regulatory subunit, ARID1A. Indeed, we found that endogenous ARID1A and MED12 bound to each other in human and murine IECs, confirming our proteomic analysis (Figure 5, E and F, and Supplemental Figure 9F). While ARID1A phosphorylation has been previously reported (46), the responsible kinase and functional implications are not known. To determine whether Mediator kinases directly phosphorylate ARID1A, we expressed and purified ARID1A protein fragments that were WT or harbored mutations at the putative CDK8/19 phosphosites (Ser365, Ser696, Ser699, Ser703, Ser716). ARID1A protein was then subjected to an in vitro kinase assay using a reconstituted human CDK8 kinase module (CDK8, MED12, and CCNC). We found site-specific CDK8-dependent phosphorylation of ARID1A at Ser365, but not Ser696–Ser716. Importantly, protein levels were equivalent for the tested WT and mutant forms of ARID1A (Figure 5G). Cumulatively, these data show that the CDK8/19 kinase module (MED12 and CDK8) associates with and phosphorylates components of the SWI/SNF complex in the context of the intestinal epithelium.

### Deletion of Mediator kinases leads to perturbed enhancer activity in a distinct subset of ARID1A-regulated genes.

**Figure 6:**
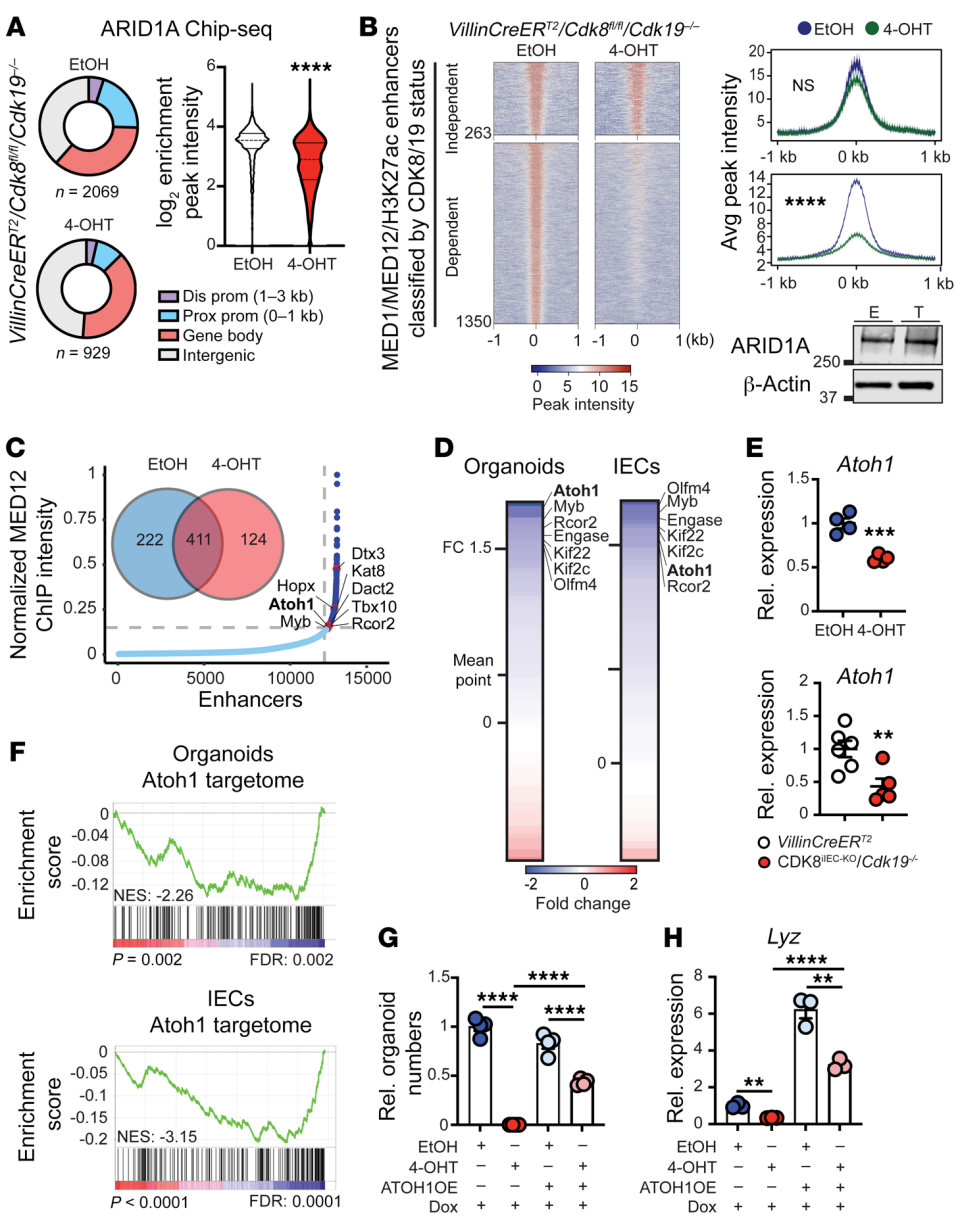
Integrated genomic-transcriptomic profiling identifies lineage-specifying transcription factors directly regulated by Mediator kinase. (**A**) Pie chart showing ARID1A binding distribution and violin plot for ARID1A peak intensity in *VillinCreER^T2^*/*Cdk8^fl/fl^*/*Cdk19^–/–^* organoids 7 days after EtOH or 4-OHT treatment. Total number of peaks for each condition is indicated. Mann-Whitney test. (**B**) Heatmap for ARID1A binding regions and average ChIP peak intensity plots at MED1/MED12/H3K27ac enhancers in EtOH- and 4-OHT–treated *VillinCreER^T2^*/*Cdk8^fl/fl^*/*Cdk19^–/–^* organoids. Immunoblot for total ARID1A protein in *VillinCreER^T2^*/*Cdk8^fl/fl^*/*Cdk19^–/–^* organoids 7 days after EtOH (E) or 4-OHT (T) treatment. (**C**) Hockey stick plot showing MED12-associated enhancers and super-enhancers and Venn diagram of super-enhancers in EtOH- and 4-OHT–treated *VillinCreER^T2^*/*Cdk8^fl/fl^*/*Cdk19^–/–^* organoids. (**D**) Gene expression as fold change of genes associated with MED1/MED12-associated super-enhancers lost after ablation of CDK8/19. Mean point represents average fold change across all MED1/MED12-associated SE-associated genes. (**E**) qRT-PCR for *Atoh1* in *VillinCreER^T2^*/*Cdk8^fl/fl^*/*Cdk19^–/–^* organoids 7 days after EtOH and 4-OHT treatment and IECs with the indicated genotypes. Unpaired 2-tailed *t* test. (**F**) GSEA for small intestinal ATOH1 target genes in *VillinCreER^T2^*/*Cdk8^fl/fl^*/*Cdk19^–/–^* organoids 7 days after EtOH and 4-OHT treatment and IECs with the indicated genotypes. (**G**) Relative organoid numbers at passage 3 after EtOH and 4-OHT treatment of parental *VillinCreER^T2^*/*Cdk8^fl/fl^*/*Cdk19^–/–^* organoids and *VillinCreER^T2^*/*Cdk8^fl/fl^*/*Cdk19^–/–^* organoids containing a doxycycline-inducible (Dox-inducible) ATOH1 overexpression construct after Dox pulsing for 24 hours following 48 hours in the absence of Dox. Representative data from 3 independent experiments. ATOH1OE, ATOH1 overexpression construct. One-way ANOVA with Tukey’s multiple-comparison test. (**H**) qRT-PCR for *Lyz* after Dox pulsing for 24 hours followed by 48 hours without Dox of EtOH- and 4-OHT–treated parental and ATOH1-overexpression *VillinCreER^T2^*/*Cdk8^fl/fl^*/*Cdk19^–/–^* organoids. Representative data from 2 independent experiments. ATOH1OE, ATOH1 overexpression construct. One-way ANOVA with Tukey’s multiple-comparison test. Data represent mean ± SEM. **P ≤ 0.01, ***P ≤ 0.005, ****P ≤ 0.001.

ARID1A has been intimately linked to intestinal differentiation programs and colon carcinogenesis (12, 47). As both Mediator and SWI/SNF have been implicated in enhancer-mediated transcriptional control, we performed ChIP-Seq to investigate their genomic occupancy in intestinal organoids in the presence and absence of CDK8/19. Firstly, ChIP-Seq for RNA polymerase II revealed that overall RNA polymerase II binding and distribution were unchanged after CDK8/19 deletion (Supplemental Figure 10A), indicating that, in contrast to cancer cells (40), loss of CDK8/19 does not affect global gene regulation in normal intestine. Similarly, ChIP-Seq analysis showed no change in the amount or distribution of H3K27ac sites, MED1, and MED12 upon loss of CDK8/19 (Supplemental Figure 10, B–D). In contrast, ARID1A genomic occupancy was both reduced (log_2_FC = 1.15; *P <* 0.001) and showed changes in binding distribution to enhancer and promoter regions after CDK8/19 ablation (Figure 6A). Given the critical role of SWI/SNF in enhancer-mediated transcription, we then categorized MED1/MED12/H3K72ac–defined enhancers based on whether they were lost (*n =* 1,350) or retained (*n =* 263) upon CDK8/19 ablation. Strikingly, we observed a near-complete loss of ARID1A binding to enhancers that were dependent on the presence of CDK8/19 (Figure 6B and Supplemental Table 6). Importantly, overall ARID1A levels were not changed in CDK8/19–deficient intestinal organoids. Overall, these data show that Mediator kinases are required for ARID1A genomic binding, and are consistent with our proteomic studies suggesting a regulatory role for CDK8/19 in SWI/SNF activity.

Enhancers and, in particular, super-enhancer (SE) elements have been intimately linked with cell fate specification by their enabling of master transcription factors (TFs) to bind and control cell type–specific transcriptional programs (48). Given the critical role of CDK8/19 in secretory intestinal cell specification, we sought to map the super-enhancer gene regulatory landscape of the intestinal epithelium in the presence and absence of CDK8/19. We found 633 MED12-defined SE regions, of which 222 were lost upon CDK8/19 deletion. We then used the Genomic Regions Enrichment of Annotations Tool (GREAT) (49) to identify *cis*-regulated genes associated with MED12-defined SE elements. From a total of 92 SE *cis*-associated genes, we found 8 TFs and developmental regulator genes associated with these SE elements, including genes previously implicated in ISC maintenance and differentiation: *ATOH1* (50), *MYB* (51), and *HOPX* (52) (Figure 6C). To determine whether the expression of these SE-associated genes was dependent on CDK8/19, we investigated their expression in intestinal organoids and IECs lacking CDK8/19. Gene expression across all 92 SE-associated genes was globally downregulated, with the majority of genes below the 0 point of log_2_FC (Figure 6D). In particular, *ATOH1*, a critical lineage specification TF of the intestinal secretory pathway, was one of the most significantly downregulated SE-associated genes upon CDK8/19 loss (Figure 6D). These data pinpoint specific SE-associated genes that may require CDK8/19 for their appropriate expression and, as such, offer potential effector proteins that are necessary for intestinal differentiation.

### CDK8 and CDK19 control secretory lineage differentiation by regulating Atoh1 expression.

Basic helix-loop-helix transcription factor ATOH1 is the master regulator of intestinal secretory cell differentiation (1). ATOH1 overexpression results in expansion of secretory cells (53), and conversely, deletion of ATOH1 results in the loss of intestinal secretory cells (50, 54), similar to the phenotype we observe in the intestine upon loss of the Mediator kinases. To confirm whether ATOH1 is a target of CDK8/19, we performed qRT-PCR for *Atoh1* expression on both organoids and IECs after CDK8/19 ablation. Indeed, *Atoh1* expression was significantly reduced in both IECs and organoids after CDK8/19 ablation (Figure 6E). Consistently, there was a significant reduction in the small intestinal ATOH1 target gene expression (ATOH1 targetome) (55) in both CDK8/19–ablated small intestinal IECs (normalized enrichment score [NES] = –3.15, *P <* 0.001) and organoids (NES = –2.26, *P =* 0.002) (Figure 6F). Consistent with a kinase-dependent effect, the expression of both ATOH1 itself and its target genes was significantly reduced in CDK8^iIEC-KO^/*Cdk19^D173A/–^* IECs and organoids (Supplemental Figure 10, E–H). We then tested whether restoration of ATOH1 could rescue the growth defect in CDK8/19-depleted intestinal organoids. To do so, we stably transduced *VillinCreER^T2^*/*Cdk8^fl/fl^*/*Cdk19^–/–^* organoids with a lentivirus encoding a doxycycline-regulated ATOH1 construct and confirmed its inducible expression (Supplemental Figure 10, I and J). Strikingly, exogenous expression of ATOH1 in CDK8/19–deficient organoids restored expression of PC markers and *Wnt3* and partially rescued the growth defect in Mediator kinase–depleted organoids (Figure 6, G and H, and Supplemental Figure 10K). Since overexpression of ATOH1 at least partially restores Wnt3 expression, this suggests that the Mediator kinase/ATOH1 axis is upstream of PC differentiation and Wnt3 expression. ATOH1 overexpression also restored expression of other secretory cell types in CDK8/19–deficient organoids, including goblet cells (*Muc2*) and tuft cells (*Dclk1*) (Supplemental Figure 10, L and M).

Notch signaling and its downstream transcription factor HES1 are known to repress Atoh1 transcription and limit secretory pathway differentiation in a process known as lateral inhibition (1). We then asked whether CDK8/19–mediated Atoh1 regulation is Notch pathway dependent. To assess whether Mediator kinases control secretory cell differentiation as part of the Notch/HES1/ATOH1 axis, CDK8/19–deficient organoids (3 days after 4-OHT) were treated with the γ-secretase inhibitor DAPT for 3 days to inhibit Notch signaling. We found that CDK8/19-depleted organoids subjected to Notch inhibition still retained the ability to upregulate a number of secretory lineage cell markers to an extent comparable to that seen in DMSO-treated controls (e.g., 30-fold [CDK8/19 knockout] vs. 37-fold [control] for *Lyz* expression) (Supplemental Figure 11A). Consistent with this, *Hes1* mRNA expression was unchanged in CDK8/19–deficient IECs, slightly elevated in CDK8/19–depleted organoids, and unchanged at the protein level (Supplemental Figure 11, B and C). Likewise, a HES1-associated super-enhancer was unaffected by loss of Mediator kinases (Supplemental Figure 11D). Collectively, these data provide evidence that the Notch pathway and its downstream effector HES1 are intact in CDK8/19-depleted organoids and, moreover, imply that Mediator kinases may regulate ATOH1 and secretory lineage differentiation independently of Notch/HES1 signaling.

### CDK8 and CDK19 control Atoh1 expression by regulating a distinct ARID1A-defined enhancer.

**Figure 7:**
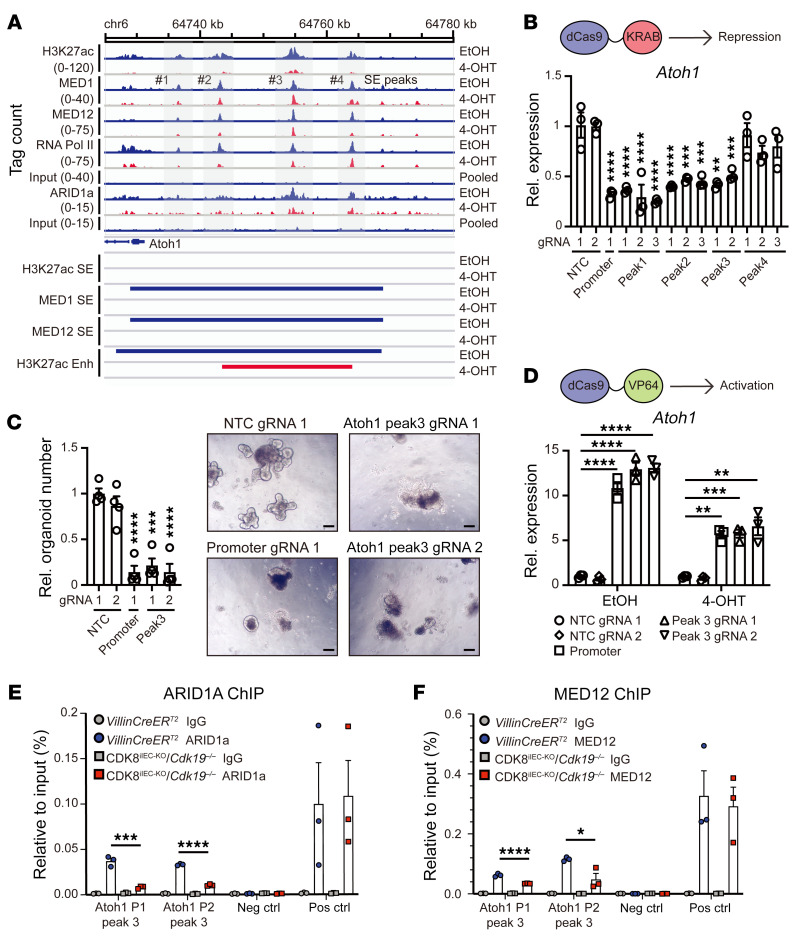
Genomic mapping of a Mediator/SWI-SNF–co-defined enhancer element critical for secretory cell lineage specification. (**A**) Genome tracks for H3K27ac, MED1, MED12, ARID1A, and RNA polymerase II (RNA Pol II) ChIP-Seq signals in the vicinity of the *Atoh1* locus in *VillinCreER^T2^*/*Cdk8^fl/fl^*/*Cdk19^–/–^* organoids 7 days after EtOH or 4-OHT treatment. Pooled input is shown as a control. Locations of the 4 super-enhancer defined peaks are marked. Gray-shaded areas depict ATOH1-associated super-enhancer peaks. (**B**) *Atoh1* qRT-PCR in C57BL/6 small intestinal organoids harboring nuclease-dead dCas9-KRAB and transduced with the indicated guide RNAs (gRNAs). gRNA-targeted regions are marked as NTC, nontargeting control; Peaks 1–4, the Atoh1 super-enhancer peaks shown in **A** above. One-way ANOVA with Dunnett’s multiple-comparison test. (**C**) Bar plot shows organoid number of C57BL/6 organoids expressing dCas9-KRAB transduced with gRNAs targeting the indicated regions (Peak 3, Atoh1 super-enhancer peak 3). Images show organoids from the indicated conditions. One-way ANOVA with Dunnett’s multiple-comparison test. Scale bars: 100 μm. (**D**) *Atoh1* qRT-PCR in *VillinCreER^T2^*/*Cdk8^fl/fl^*/*Cdk19^–/–^* organoids expressing dCas9-VP64 72 hours after transduction with gRNAs targeting ATOH1 promoter, ATOH1 super-enhancer peak 3, and NTC control. Representative data from 3 independent experiments. One-way ANOVA with Dunnett’s multiple-comparison test. (**E**) ChIP-qPCR for Atoh1 super-enhancer peak 3 after ARID1A or IgG immunoprecipitation in primary IECs from *VillinCreER^T2^* and CDK8^iIEC-KO^/*Cdk19^–/–^* mice 21 days after the first tamoxifen injection. P1, primer 1; P2, primer 2; Peak 3, Atoh1 super-enhancer peak 3. (**F**) ChIP-qPCR for *Atoh1* super-enhancer peak 3 after MED12 or IgG immunoprecipitation in primary IECs from *VillinCreER^T2^* and CDK8^iIEC-KO^/*Cdk19^–/–^* mice 21 days after the first tamoxifen injection. Data represent mean ± SEM. *P ≤ 0.05, **P ≤ 0.01, ***P ≤ 0.005, ****P ≤ 0.001.

Given the Notch-independent mechanism of ATOH1 regulation by CDK8/19, we investigated whether the ATOH1-associated SE element identified through our ChIP-Seq studies may provide an alternative mode of regulating *Atoh1* expression. ChIP-Seq peak density plots for the *Atoh1* locus revealed 4 separate peaks of H3K27ac, MED1, and MED12 binding about 10–30 kb downstream of the *Atoh1* locus within the MED1/MED12–defined super-enhancer. Interestingly, ARID1A binding was found specifically at peaks 3 and 4 and only in the presence of CDK8/19. In line with reduced activity from this SE element, the H3K27ac mark and RNA polymerase II binding were greatly diminished alongside reduced ATOH1 expression levels (Figure 7A). To assess the functional importance of this ATOH1-associated super-enhancer (peaks 1–4), we used CRISPR interference (dCas9-KRAB repressor) to specifically dampen Atoh1-associated enhancer-mediated transcriptional output in C57BL/6 WT intestinal organoids. Targeting dCas9-KRAB to the *Atoh1* promoter using specified sgRNAs resulted in significantly reduced Atoh1 expression (Figure 7B), confirming the assay’s functionality. Interestingly, silencing of peak 1, peak 2, and peak 3, but not peak 4, resulted in significant *Atoh1* downregulation (Figure 7B), suggesting that these peaks define functional enhancers that mediate ATOH1 expression in the intestine. Consistently, organoids with CRISPR-targeted suppression of the *Atoh1* promoter and ATOH1 super-enhancer peaks 1–3 displayed significant growth defects (Figure 7C), confirming that these enhancers are functionally critical for ATOH1 expression and organoid viability.

Our previous data indicate that the SWI/SNF complex may act as an important mediator of CDK8/19 function in the intestine. To test whether the ARID1A-bound site enhancer region is critical for ATOH1 transcription, we used CRISPR activation (CRISPRa) to direct a strong transcriptional activator (dCas9-VP64) to the ARID1A-defined *Atoh1*-associated enhancer (peak 3). The system was confirmed using a guide RNA targeting the *Atoh1* promoter, which showed robust activation of *Atoh1* transcription (Figure 7D). Strikingly, CRISPRa targeted activation of the ARID1A-defined enhancer (peak 3) showed similar induced *Atoh1* expression in CDK8^iIEC-KO^/*Cdk19^–/–^* organoids compared with negative controls (Figure 7D). Interestingly, CRISPRa-induced ATOH1 expression from peak 3 was lower in CDK8/19–deficient organoids compared with controls. Lastly, to test whether this regulatory mechanism applies in vivo, we assessed ARID1A and MED12 binding at the Atoh1 super-enhancer peak 3 in CDK8/19–deficient IECs by ChIP-qPCR. Using 2 primer pairs targeting peak 3 of this enhancer, we found that ARID1A and MED12 binding at the Atoh1 super-enhancer peak 3 was significantly reduced in CDK8^iIEC-KO^/*Cdk19^–/–^* mice compared with *VillinCreER^T2^* IEC controls (Figure 7, E and F). These results show that the Mediator kinase module is a direct regulator of ATOH1 expression and secretory cell differentiation in vivo as well. Taken together, these data define a CDK8/19–dependent, enhancer-mediated mechanism of *Atoh1* transcription that involves the interplay of both MED12-Mediator and SWI/SNF complex components.

## Discussion

Using genetic mouse models combined with transcriptomic, epigenetic, and phospho-proteomic profiling, we identify a kinase-dependent function for the Mediator kinases CDK8 and CDK19 in intestinal secretory cell type differentiation. Importantly, we find that CDK8 and CDK19 act in a functionally redundant manner to control transcriptional output and differentiation. We show that the Mediator complex interacts with the SWI/SNF complex members and is required for proper ARID1A binding across MED1/12–defined intestinal enhancer regions, suggesting that the Mediator kinase complex may act beyond transcriptional regulation to regulate chromatin accessibility.

Previous studies have suggested that CDK8 and its paralog CDK19 have nonredundant roles as transcriptional regulators (23, 27). Moreover, other studies have identified both kinase-dependent and -independent functions for CDK8/19 (25, 26, 56). Herein, we show that CDK8 and CDK19 have redundant and kinase-dependent functions in regulating transcription in normal intestinal homeostasis. This is consistent with our own previous work in CRC, where we found that single gene ablation of CDK8 or CDK19 has minor effects on gene transcription and functional output. Intriguingly, CDK8/19 loss produced broad effects in the cancer state compared with normal tissue. While CDK8/19 ablation led to global loss of transcription and enhancer deposition (40), depletion of CDK8/19 in the normal intestine was marked by enhancer perturbation and transcriptional loss of only a distinct subset of lineage-specific genes. These results suggest divergent, yet kinase-dependent, functions for Mediator kinases in regulating transcription in the normal versus oncogenic states. We speculate that these differences may be attributed to cell type–specific functions of Mediator kinases or the fact that other studies have been performed using transformed cell lines. Intestinal epithelial cells are generally characterized by broadly permissive chromatin (10) combined with a set of unique active enhancers allowing the activity of cell lineage–specific TFs as essential determinants of intestinal cell lineage identities (57). We provide evidence that CDK8 and CDK19 act within this framework and are required to maintain the activity of super enhancers important for TFs regulating intestinal epithelial cell identity.

Our study provides a comprehensive phospho-proteomic analysis of Mediator kinase module interactors and potential phosphorylation substrates. Remarkably, 3 of the top 9 phosphorylated targets were SWI/SNF components, including ARID1A, a critical tumor suppressor gene (47, 58–60). Intriguingly, CDK8/19 ablation in the intestine phenocopies defects in secretory progenitor differentiation seen upon ARID1A loss in the intestine, although the phenotype of ARID1A-deficient mice is more severe with additional loss of intestinal stem cells (12). Curiously, while we identify a number of CDK8/19 kinase substrates that are consistent with a previous phospho-proteomic analysis in a CRC cell line (e.g., CHD4, MED12, MED13; ref. 44), the SWI/SNF components that we identified were only found in the context of the normal intestine. It is interesting to note that the cell line used in the previous study, HCT116 cells, harbors a SMARCA4 mutation (L1149P) predicted to compromise the SWI/SNF complex (45). This may explain the context-specific regulation of SWI/SNF by Mediator kinases in the normal intestine compared with the cancer state. Given the ubiquitous nature of both Mediator and SWI/SNF complexes, it will be of great interest to broaden our understanding of how Mediator kinases regulate SWI/SNF activity in other tissues as well.

Strikingly, we show that the MED12-Mediator complex interacts with most components of the SWI/SNF complex, suggestive of a broader association between Mediator and SWI/SNF complexes. Importantly, SWI/SNF enhancer occupancy was CDK8/19 dependent, as we observed a widespread reduction of ARID1A binding across the genome and particularly at MED1/12–defined enhancers after CDK8/19 loss. While the exact nature of these interactions remains to be determined, it is exciting to speculate that Mediator complex function may extend beyond its canonical role in transcription to control of higher-order chromatin accessibility via its ability to regulate SWI/SNF activity.

The mechanism of lineage specification in the intestinal tract requires the hierarchical activation of discrete transcriptional programs. Previous work has demonstrated that Notch inhibition, HES1 deletion, and overexpression of ATOH1 result in overabundance of secretory cells, while ATOH1 deletion leads to a loss of secretory cells (50, 54, 61–63). While the Notch pathway and its role in regulating ATOH1 via HES1 have been extensively studied, the cell-intrinsic mechanisms through which ATOH1 is expressed have remained elusive. Our work directly implicates Mediator kinases as cell-intrinsic regulators of enhancer-mediated ATOH1 transcription.

Importantly, pharmacological inhibition of Notch overcame CDK8/19–mediated ATOH1 suppression, highlighting that the Notch/HES1 pathway is still intact in the absence of CDK8/19. Consistent with this, previous reports show that deletion of either ATOH1 or ARID1A has a more drastic impact on the intestinal secretory cell compartment compared with CDK8/19 ablation (12, 50, 54). This highlights the diverse mechanisms that contribute to fine-tuning of the expression of ATOH1 and subsequent production of secretory cell types.

In our study, CDK8/19–depleted intestinal cells did not show significant changes in Wnt/β-catenin–mediated transcriptional output. Consistent with this, Wnt stimulation did not rescue the effects of CDK8/19 loss on secretory type differentiation. Hence, we propose that the requirement of CDK8/19 for secretory cell type differentiation is independent of the Wnt/β-catenin pathway. This contrasts with several previous studies, using genetic and pharmacological inhibitors, which have reported a direct role for CDK8/19 in mediating Wnt/β-catenin transcription in the context of CRC. Further studies comparing CDK8/19 activity in the normal and neoplastic intestine will be helpful to delineate the contextual differences of CDK8/19 activity in the gut. Taken together, these findings highlight the ability of signal-driven transcriptional effectors and chromatin-mediated regulators to act as molecular rheostats of intestinal secretory lineage specification.

### Limitations of the study.

Forced expression of ATOH1 from a constitutive promoter did not completely rescue the phenotype of CDK8/19–deficient organoids. While the incompleteness of the rescue effect may be related to technical challenges of expressing the appropriate amount of ATOH1 in the correct cell type, we also cannot rule out that CDK8/19–mediated secretory cell type differentiation may also involve ATOH1-independent mechanisms. Our study was conducted in intestinal epithelial cells, which enables us to place the function of Mediator kinases within the context of one of the most well-studied tissue homeostatic mechanisms. While our conclusions, with regard to SWI/SNF regulation, are broadly applicable to other tissue types, a proper analysis of Mediator kinase–SWI/SNF interaction in additional tissue systems would be important. Furthermore, while we show that the Mediator kinase module associates with SWI/SNF, it would be important to better understand whether these interactions occur in the context of the core Mediator complex or are independent of core Mediator function.

## Methods

A complete description of the methods is provided in Supplemental Methods.

### Data availability.

RNA-Seq and ChIP-Seq data were deposited in the NCBI’s Gene Expression Omnibus database (GEO GSE191124). Mass spectrometry proteomics data generated in this study were deposited in ProteomeXchange via the PRIDE (https://www.ebi.ac.uk/pride/) partner repository with the data set identifiers PXD030212 and PXD030256. This paper does not report original code.

### Statistics.

Data shown in column graphs represent the mean ± SEM, as indicated in the figure legends. To determine the group size necessary for adequate statistical power, power analysis was performed using preliminary data sets. When data fulfilled the criteria for Gaussian distribution, unpaired 2-tailed Student’s *t* test was performed; otherwise the nonparametric Mann-Whitney test was chosen. Significance of cell signatures within gene clusters was calculated with Fisher’s exact test followed by Bonferroni’s correction for *P* value multiple testing correction. *P ≤ 0.05; **P ≤ 0.01; ***P ≤ 0.005; ****P ≤ 0.001. Statistical analysis was performed with Prism 9 (GraphPad) or Excel (Microsoft) software.

### Study approval.

Human samples were obtained from patients undergoing surgery at Monash Health with written informed consent and under approved ethics (Victorian Pancreatic Cancer Biobank, HREC/15/MonH/117, Monash Health). All animal work was conducted in accordance with the National Health and Medical Research Council and Monash Animal Research Platform guidelines. All procedures were approved by the Monash Medical Centre Animal Ethics Committee under license MMCA/2017/04, Monash University.

## Author contributions

MVD and RF conceived the study, performed experiments, analyzed the data, and wrote the manuscript. DZ designed and performed experiments and analyzed the data. HKC, MP, ML, SK, TGB, DS, and MS performed and analyzed experiments. MVD and DZ performed animal experiments. XS and HG performed bioinformatics analysis. ML and MS performed qPCRs and evaluated cell lineages on tissue sections. TCCLKS, PF, and RJD performed proteomics analysis. DC provided human small intestinal tissues. All authors approved the manuscript. MVD and DZ share co–first authorship due to their sizeable contributions. The order of first authors was decided based on MVD’s role in coordinating the work of the team and on the length of involvement in the study.

## Supplementary Material

Supplemental data

Supplemental table 1

Supplemental table 2

Supplemental table 3

Supplemental table 4

Supplemental table 5

Supplemental table 6

Supplemental table 7

## Version 1 08/25/2022

In-Press Preview

## Version 2 10/17/2022

Electronic publication
