# Supplementary material for: CDK8 and CDK19 regulate intestinal differentiation and homeostasis via the chromatin remodeling complex SWI/SNF
Source: J Clin Invest. 2022 Oct 17;132(20):e158593. doi: 10.1172/JCI158593 (PMC9566890; doi:10.1172/JCI158593)
Supplement: Supplemental data [file jci-132-158593-s144.pdf]

Table Legend

Table S1. DEGs in organoids and IECs
DEGs (up- and down) from RNA-sequencing (whole transcriptome) studies using FC cut-off ±1.5, FDR<0.05. DEGs (up- and down). Each tab indicates a separate comparison as described in the table.

Table S2. RNA-seq Timecourse
Cluster analysis of genes based on their expression pattern in VillinCreER^{T2}/*Cdk8*$^{fl/fl}$/*Cdk19*$^{-/-}$ organoids on days 3, 5, 7 and 10 after EtOH and 4-OHT treatment.

Table S3. MED1IP-LC-MS
Quantified proteins after MED1 or IgG IP followed by LC-MS/MS in EtOH and 4-OHT treated in VillinCreER^{T2}/*Cdk8*$^{fl/fl}$/*Cdk19*$^{-/-}$ organoids on day 7. All non-nuclear proteins were excluded.

Table S4. Phosphoproteomics
Identified Phosphorylation events in EtOH and 4-OHT treated in VillinCreER^{T2}/*Cdk8*$^{fl/fl}$/*Cdk19*$^{-/-}$ organoids on day 5 after treatment. Sig P-sites were determined by filtering for Log2FC <-1 and a p-value <0.05 and further filtered for nuclear proteins.

Table S5. MED12IP-LC-MS
Quantified proteins after MED12IP (vs IgG) in EtOH treated VillinCreER^{T2}/*Cdk8*$^{fl/fl}$/*Cdk19*$^{-/-}$ organoids. Proteins were filtered for nuclear proteins and a p-value of <0.05.

Table S6. Chip-seq
ChIP-seq of VillinCreER^{T2}/*Cdk8*$^{fl/fl}$/*Cdk19*$^{-/-}$ organoids 7 days after EtOH and 4-OHT treatment. Enhancer intervals and associated genes for H3K27ac, MED1 and MED12. Merged regions with interval count for Arid1a in EtOH and 4-OHT treated VillinCreER^{T2}/*Cdk8*$^{fl/fl}$/*Cdk19*$^{-/-}$ organoids.

Table S7. Primer-sequences
Primer sequences used in this study.

Supplemental Figure 1

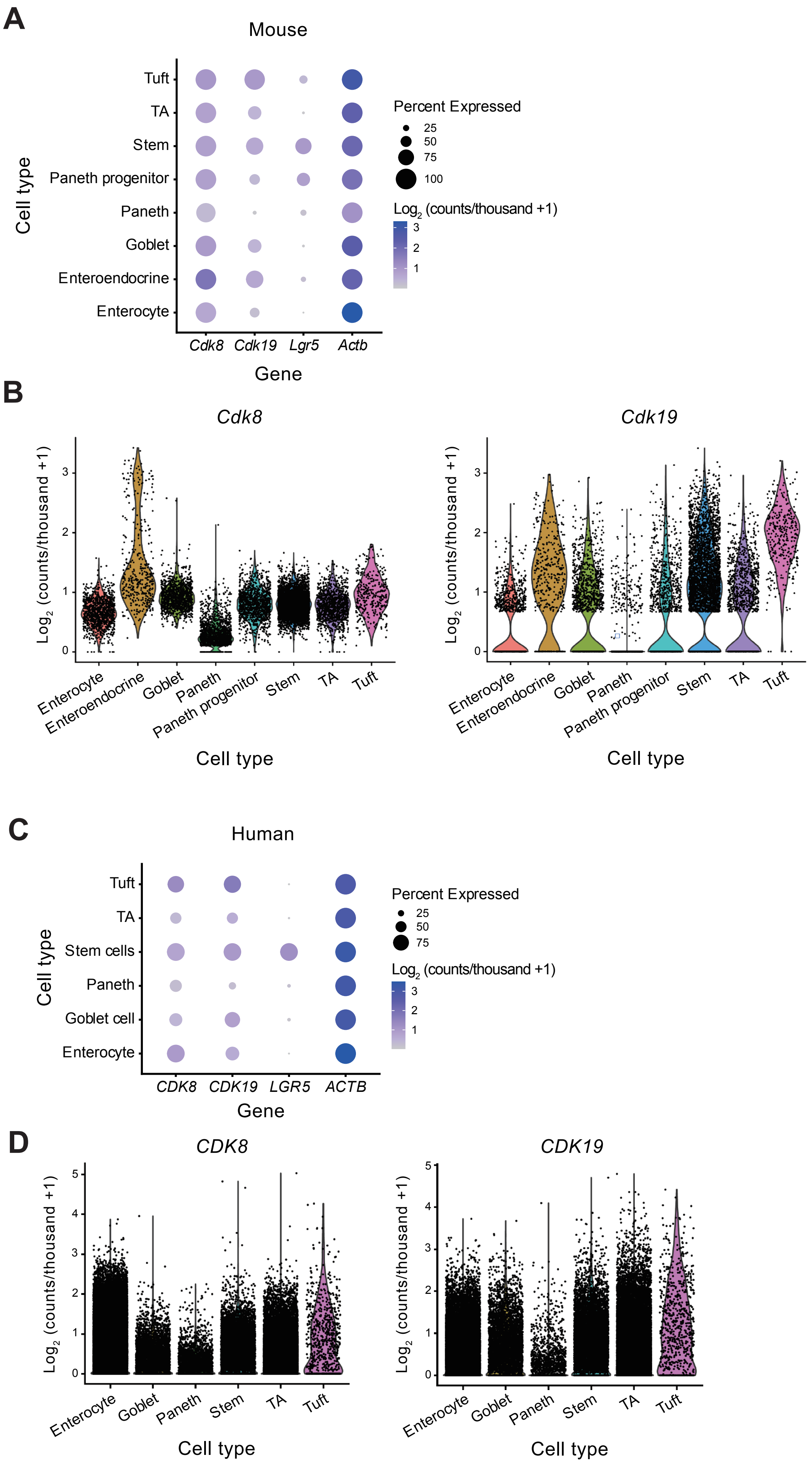

Figure S1. *In silico* analysis of CDK8 and CDK19 expression in mouse and human small intestinal cell types.

(A) Percentage and average gene expression of *Cdk8* and *Cdk19* across defined cell populations extracted from a scRNA-seq dataset of the normal mouse intestinal epithelium (Haber *et al*. Nature 2017). *Lgr5* and *Actb* are shown as examples of cell-delimited expression and ubiquitous expression patterns, respectively.

(B) Violin plots show *Cdk8* and *Cdk19* expression in murine small intestinal cell types as in panel (**A)**.

(C) Percentage and average expression of *CDK8* and *CDK19* across defined cell populations of a scRNA-seq dataset of the human intestinal epithelium (Elmentaite *et al*. Nature 2021). *LGR5* and *ACTB* are shown as examples of cell-delimited expression and ubiquitous expression patterns, respectively.

(D) Violin plots show *CDK8* and *CDK19* expression in human small intestinal cell lineages as described in panel (**C)**.

Supplemental Figure 2

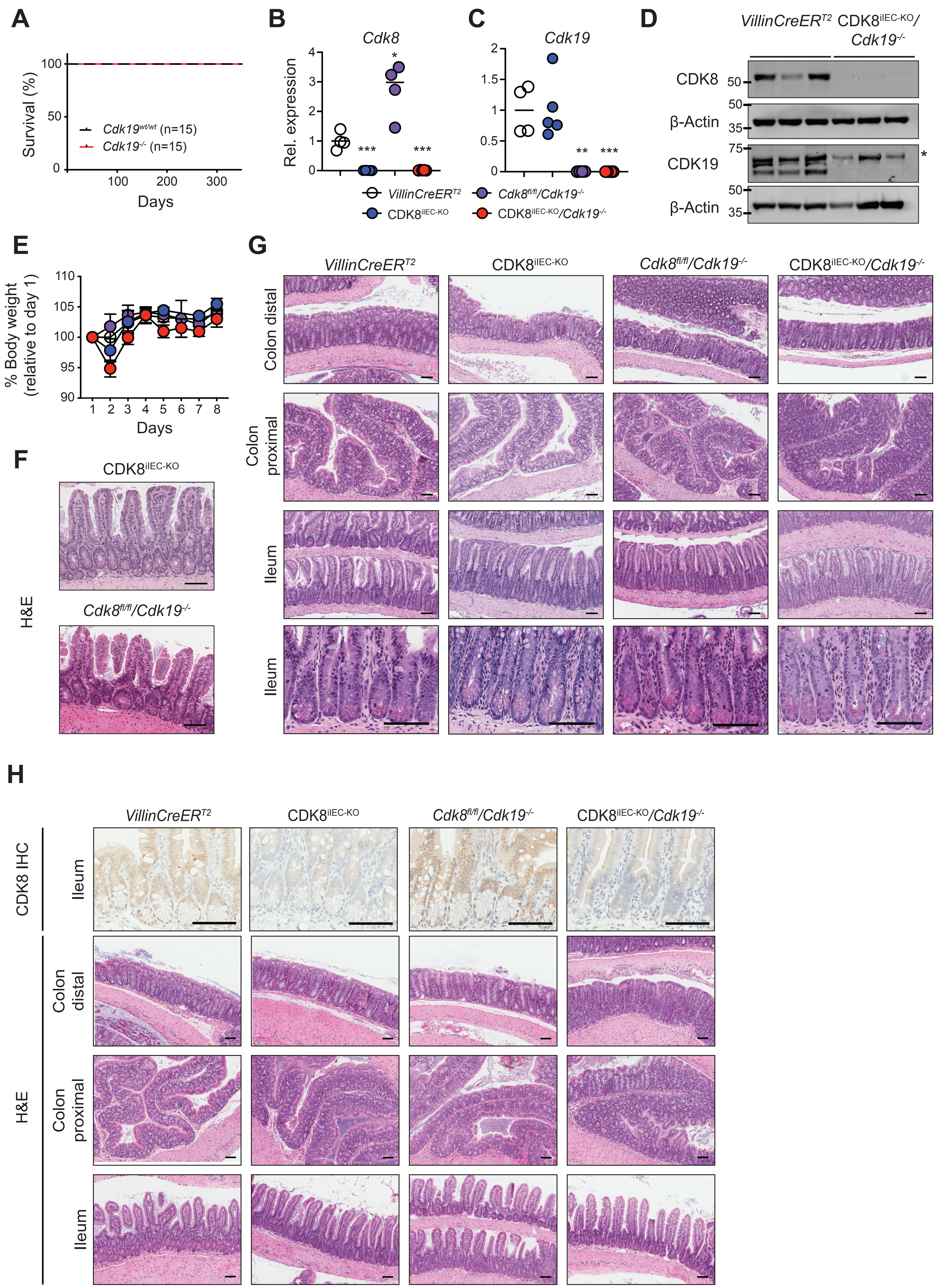

Figure S2. Cdk8 and Cdk19 are functionally redundant paralogs in intestinal homeostasis.

(A) Kaplan-Meier survival curve of *Cdk19*$^{wt/wt}$ and *Cdk19*$^{-/-}$ mice.

(B, C) qRT-PCR analysis of **(B)** *Cdk8* and **(C)** *Cdk19* expression in SI IECs from mice with the indicated genotypes 9 days after the last tamoxifen injection. Unpaired t-test used.

(D) Immunoblot of small intestinal IECs from mice with the indicated genotypes 9 days after the last tamoxifen injection. *, unspecific band.

(E) Body weight curve of tamoxifen treated (day 1-5) *VillinCreER*^{T2} or *Cdk8*$^{fl/fl}$ mice (n=4), CDK8$^{iIEC\text{-}KO}$ mice (n=4), *Cdk8*$^{fl/fl}$/*Cdk19*$^{-/-}$ mice (n=6) and CDK8$^{iIEC\text{-}KO}$/*Cdk19*$^{-/-}$ mice (n=4).

(F) Representative images of H&E-stained Ileal sections from mice with the indicated genotype 3 weeks after tamoxifen injections. Scale bars: 100µm

(G) H&E-stained colonic and ileal sections from mice with the indicated genotypes 9 days after first tamoxifen injection. Scale bars: 100µm

(H) CDK8 IHC staining and H&E staining on colonic and ileal sections of mice with the indicated genotypes 6 month after tamoxifen injections**.** Scale bars: 100µm

Data represents mean $\pm$ SEM. *$p \leq 0.05$, ** $p \leq 0.01$, ***$p \leq 0.005$, ****$p \leq 0.001$

Supplemental Figure 3

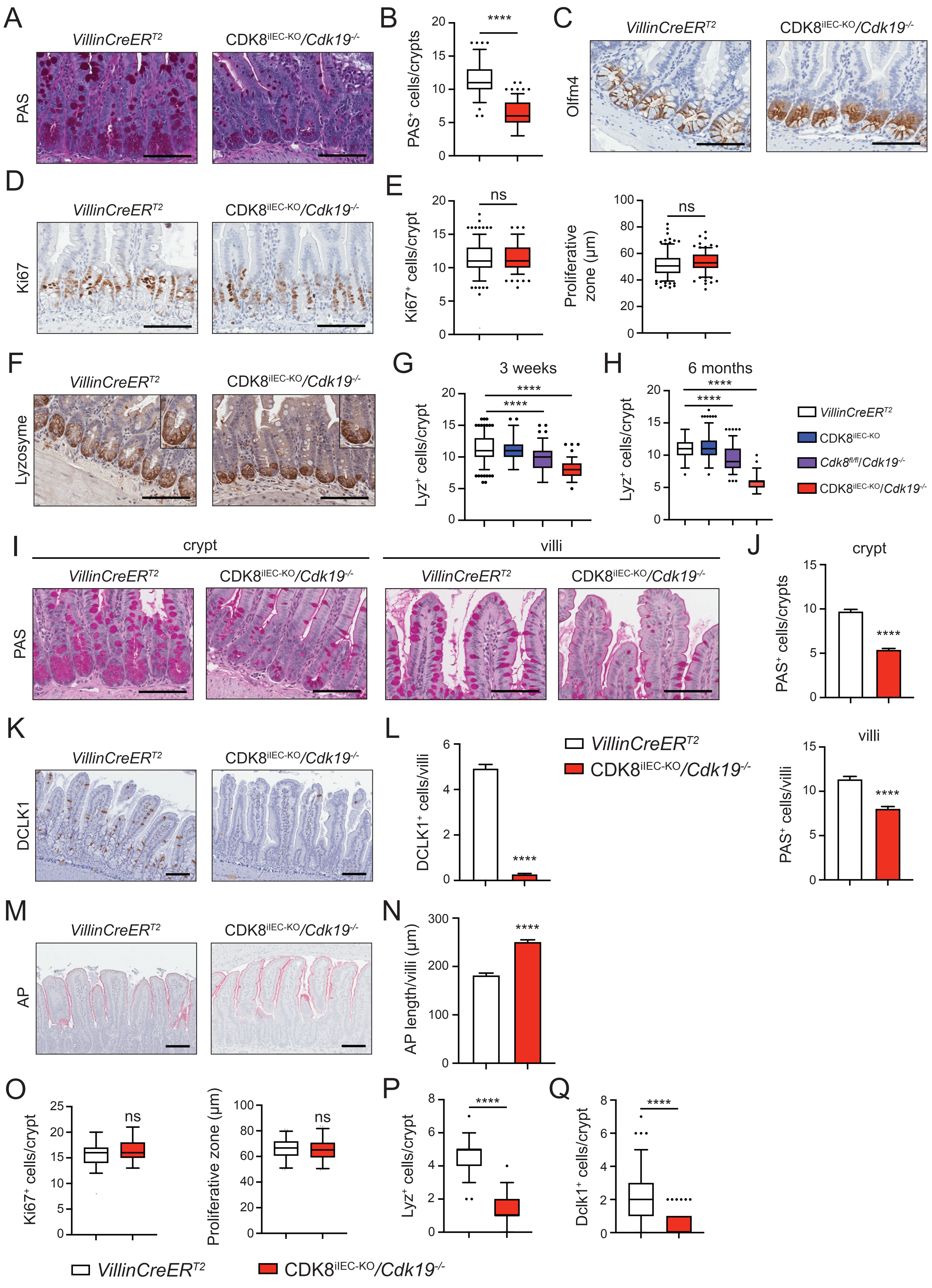

Figure S3. CDK8/19 regulate intestinal cell differentiation.

(A) Representative images of PAS-stained ileal sections from *VillinCreER*^{T2} (n=4) and CDK8$^{iIEC\text{-}KO}$/*Cdk19*$^{-/-}$ (n=5 mice) 3 weeks after tamoxifen injections. Scale bars: 100µm

(B) Quantification of PAS staining in *VillinCreER*^{T2} (n=4 mice, n=152 crypts) and CDK8$^{iIEC\text{-}KO}$/*Cdk19*$^{-/-}$ (n=5, n= 152 crypts) as box-and-whiskers (5-95 percentile).

(C) Representative images of Olfm4 IHC staining on ileal sections from *VillinCreER*^{T2} mice (n=4) and CDK8$^{iIEC\text{-} KO}$/*Cdk19*$^{-/-}$ mice (n=4). Scale bars: 100µm

(D) Representative images of Ki67 IHC staining on ileal sections from *VillinCreER*^{T2} mice (n=4) and CDK8$^{iIEC\text{-} KO}$/*Cdk19*$^{-/-}$ mice (n=4) 3 weeks after tamoxifen injections. Scale bars: 100µm

(E) Quantification of Ki67 IHC cells per crypt and length proliferative zone, indicated by Ki67^{+} cells in *VillinCreER*^{T2} mice (n=4 mice, n=200 crypts) and CDK8$^{iIEC\text{-}KO}$/*Cdk19*$^{-/-}$ mice (n=5 mice, n= 200 crypts) 3 weeks after tamoxifen injections as box-and-whiskers (5-95 percentile).

(F) Lysozyme IHC on ileal sections of mice with the indicated genotypes 6 months after tamoxifen injections. Scale bars: 100µm

(G) Quantification of Lysozyme IHC on ileal sections **3 weeks** after tamoxifen injections as box-and-whiskers (5-95 percentile). *VillinCreER*^{T2} (n=200 crypts), CDK8$^{iIEC\text{-}KO}$ mice (n=100 crypts), *Cdk8*$^{fl/fl}$/*Cdk19*$^{-/-}$ mice (n=112 crypts). Kruskal-Wallis test with Dunn's multiple comparisons test used. Sections of n≥3 mice per genotype were counted. *VillinCreER*^{T2} and CDK8$^{iIEC\text{-}KO}$/*Cdk19*$^{-/-}$ data points are the same as shown in Figure 2D and were included for comparison.

(H) Quantification of Lysozyme IHC on ileal sections **6 months** after tamoxifen injections as Box and whiskers (5-95 percentile). *VillinCreER*^{T2} (mice: n=4; crypts: n=125), CDK8$^{iIEC\text{-}KO}$ mice (mice: n=4; crypts: n=186), *Cdk8*$^{fl/fl}$/*Cdk19*$^{-/-}$ mice (mice: n=4; crypts: n=203) and CDK8$^{iIEC\text{-}KO}$/*Cdk19*$^{-/-}$ mice (mice: n=4; crypts: n=149).

(I) PAS staining on ileal sections of *VillinCreER*^{T2} (n=4) and CDK8$^{iIEC\text{-}KO}$/*Cdk19*$^{-/-}$ (n=5) 6 months after tamoxifen injections. Scale bars: 100µm

(J) Quantification of PAS staining from (**I**) as Box and whiskers (5-95 percentile). *VillinCreER*^{T2} (mice: n=4; crypt: n=153; villi: n=152) and CDK8$^{iIEC\text{-}KO}$/*Cdk19*$^{-/-}$ mice (mice: n=4; crypt: n=191, villi: n=189). Bar plot shows staining per crypt (top) or normalized to villi (bottom).

(K) IHC for DCLK1 on sections from *VillinCreER*^{T2} (n=4) and CDK8$^{iIEC\text{-}KO}$/*Cdk19*$^{-/-}$ (n=5) 6 months after tamoxifen injections. Scale bars: 100µm

(L) Quantification of DCLK1 IHC staining from (K) as Box and whiskers (5-95 percentile). *VillinCreER*^{T2} (mice: n=4; villi: n=153) and CDK8$^{iIEC\text{-} KO}$/*Cdk19*$^{-/-}$ (mice: n=4; villi: n=353).

(M) Visualization of Alkaline Phosphatase (AP) activity on sections from *VillinCreER*^{T2} (mice: n=4) and CDK8$^{iIEC\text{-}KO}$/*Cdk19*$^{-/-}$ (mice: n=4) mice. Scale bars: 100µm

(N) Quantification of AP staining from **(M)** as box-and-whiskers (5-95 percentile). *VillinCreER*^{T2} (mice: n=4; villi: n=59) and CDK8$^{iIEC\text{-} KO}$/*Cdk19*$^{-/-}$ (mice: n=4; villi: n=202)

(O) Quantification of Ki67 IHC staining of *VillinCreER*^{T2} (mice: n=4; crypts: n=140) and CDK8$^{iIEC\text{-}KO}$/*Cdk19*$^{-/-}$ (mice: n=4; crypts: n=183) mice 6 months after tamoxifen injections as box-and-whiskers (5-95 percentile).

(P) Quantification of LYZ IHC staining in the duodenum of VillinCreERT2 (mice: n=3, crypts: n=75) and CDK8$^{iIEC\text{-}KO}$/*Cdk19*$^{-/-}$ (mice: n=3; crypts: n=209) mice 6 months after tamoxifen injections as box-and-whiskers (5-95 percentile).

(Q) Quantification of DCLK1 IHC staining in the duodenum of VillinCreERT2 (mice: n=3, villi: n=165) and CDK8$^{iIEC\text{-}KO}$/*Cdk19*$^{-/-}$ (mice: n=3; villi: n= 146) mice 6 months after tamoxifen injections as box-and-whiskers (5-95 percentile).

Bar graphs represents mean ± SEM. For box-and-whisker plots, box represents 25-75th percentile, whiskers 5-95th percentile and outlying values are shown **P*≤0.05, ** *P*≤0.01, ****P*≤0.005, *****P*≤0.001

Supplemental Figure 4

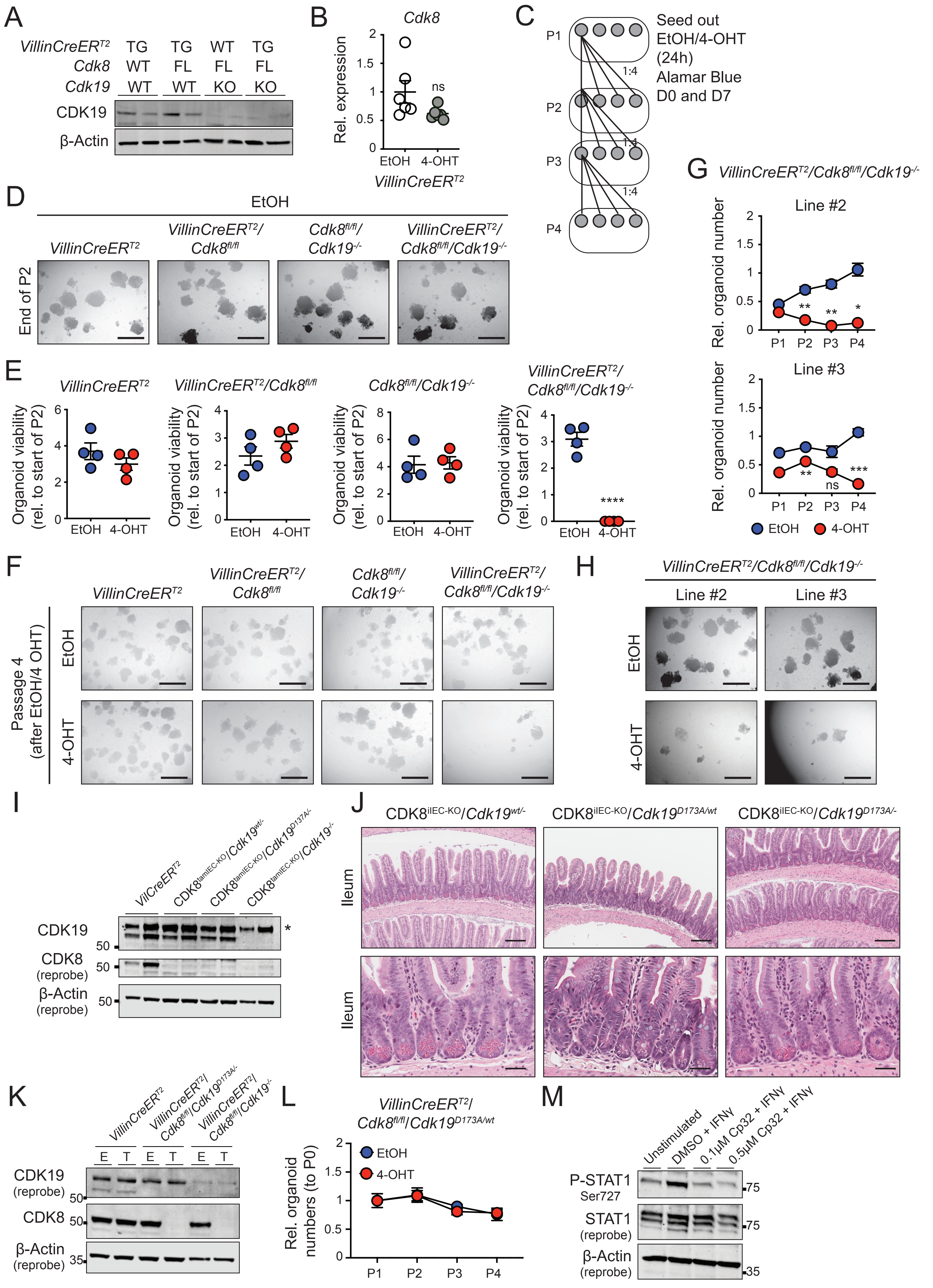

Figure S4. Mediator kinases are essential for organoid growth.
(A) Immunoblot on small intestinal organoids from mice with the indicated genotypes one week after 4-OHT treatment. TG, transgenic; FL, flox; WT, wildtype; KO, knockout.
(B) qRT-PCR analysis of *Cdk8* expression from *VillinCreER^{T2}* organoids 1 week after the indicated treatment conditions. Unpaired t-test used. Samples from two independent experiments used.
(C) Experimental layout depicting organoid treatments and passaging regime for growth assays.
(D) Representative images of organoids with the indicated genotypes, 2 passages after EtOH treatment.
(E) Plot shows viability (Alamar Blue HS) of *VillinCreER^{T2}*, *VillinCreER^{T2}/Cdk8$^{fl/fl}$*, *Cdk8$^{fl/fl}$/Cdk19$^{-/-}$*, *VillinCreER^{T2}/Cdk8$^{fl/fl}$/Cdk19$^{-/-}$* organoids at passage 2 after indicated treatments. Representative of three independent experiments shown. Unpaired t-test was used.
(F) Representative images of organoids with the indicated genotypes 4 weeks after the indicated treatments.
(G) Relative organoid numbers compared to passage 0 (50 organoids/well) from two independently *VillinCreER^{T2}/Cdk8$^{fl/fl}$/Cdk19$^{-/-}$* intestinal organoid lines over 4 passages. Treatments as indicated in legend. Two-way ANOVA with Sidak's multiple comparison test used.
(H) Representative images from the indicated intestinal organoid lines 2 passages post EtOH and 4-OHT treatment.
(I) Immunoblot on small intestinal IECS from mice with the indicated genotypes 14 days after the first tamoxifen injection. *, non-specific band.
(J) H&E-stained ileal sections from mice with the indicated genotypes 14 days after the first tamoxifen injection. Scale bars: 200µm (upper) and 50 µm (lower).
(J) Immunoblot on mouse small intestinal organoids with the indicated genotypes 7 days after EtOH and 4-OHT treatment. E, EtOH; T, 4-OHT
(K) Relative organoid numbers (compared to Passage 1) for EtOH and 4-OHT treated *VillinCreER^{T2}/Cdk8$^{fl/fl}$/Cdk19$^{D173A/wt}$* organoids for 4 Passages after treatment. Representative data of three independent experiments shown.
(L) Immunoblot on C57BL/6 mouse organoid lysates treated with DMSO or CP32 for 24 hours and stimulated with 20ng/ml interferon-γ for 45 minutes. Untreated organoids were used as control.
Data represents mean ± SEM. **P*≤0.05, ** *P*≤0.01, ****P*≤0.005, *****P*≤0.001

Supplemental Figure 5

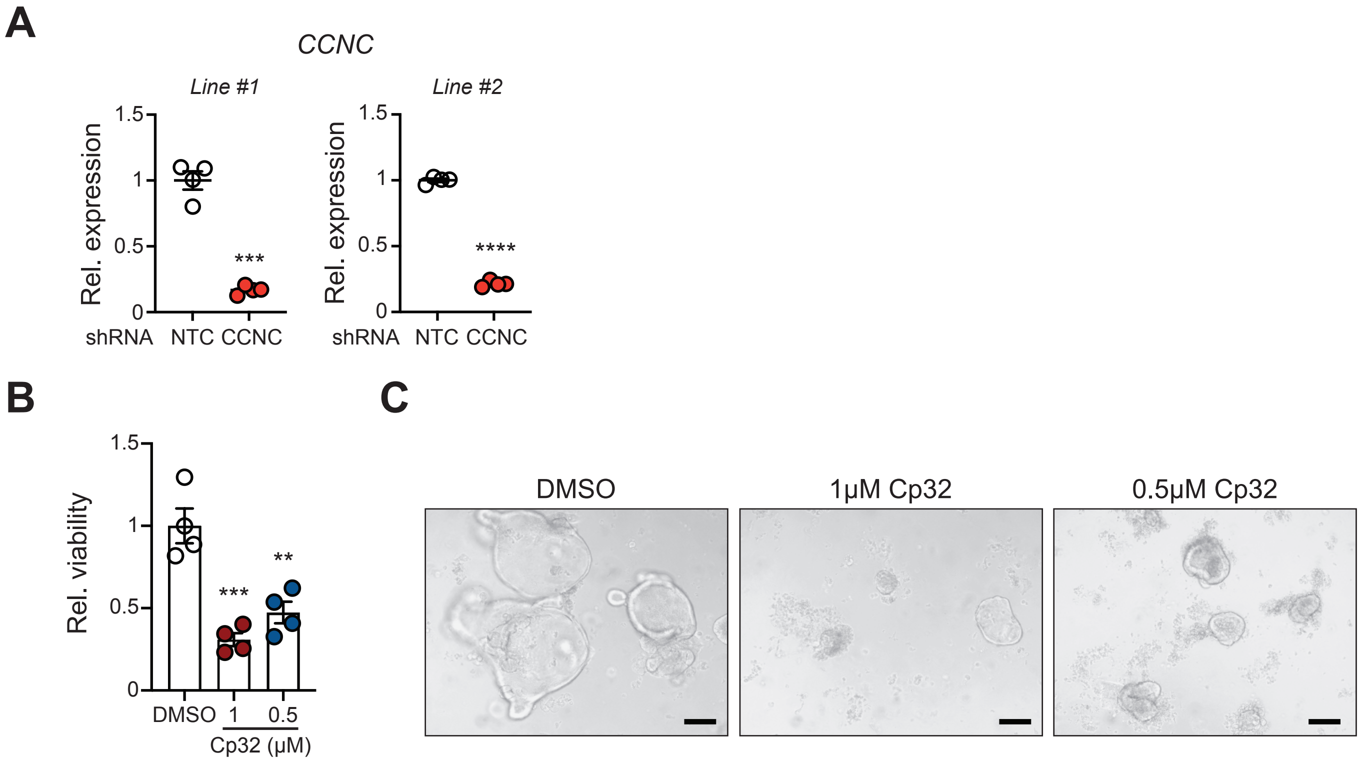

Figure S5. Mediator kinases are required for growth of human small intestinal organoids

(A) qRT-PCR analysis of *CCNC* gene expression 3 days after shRNA transduction (as indicated) in two independently derived human small intestinal organoid lines. Unpaired t-test used.

(B) Human intestinal organoid viability (Alamar Blue HS) after treatment with the indicated concentration of Cp32 or DMSO control for 2 weeks. Unpaired t-test used.

(C) Representative images from organoids in (**B**) taken 2 weeks post treatment.
Data represents mean ± SEM. *P≤0.05, ** P≤0.01, ***P≤0.005, ****P≤0.001

Supplemental Figure 6

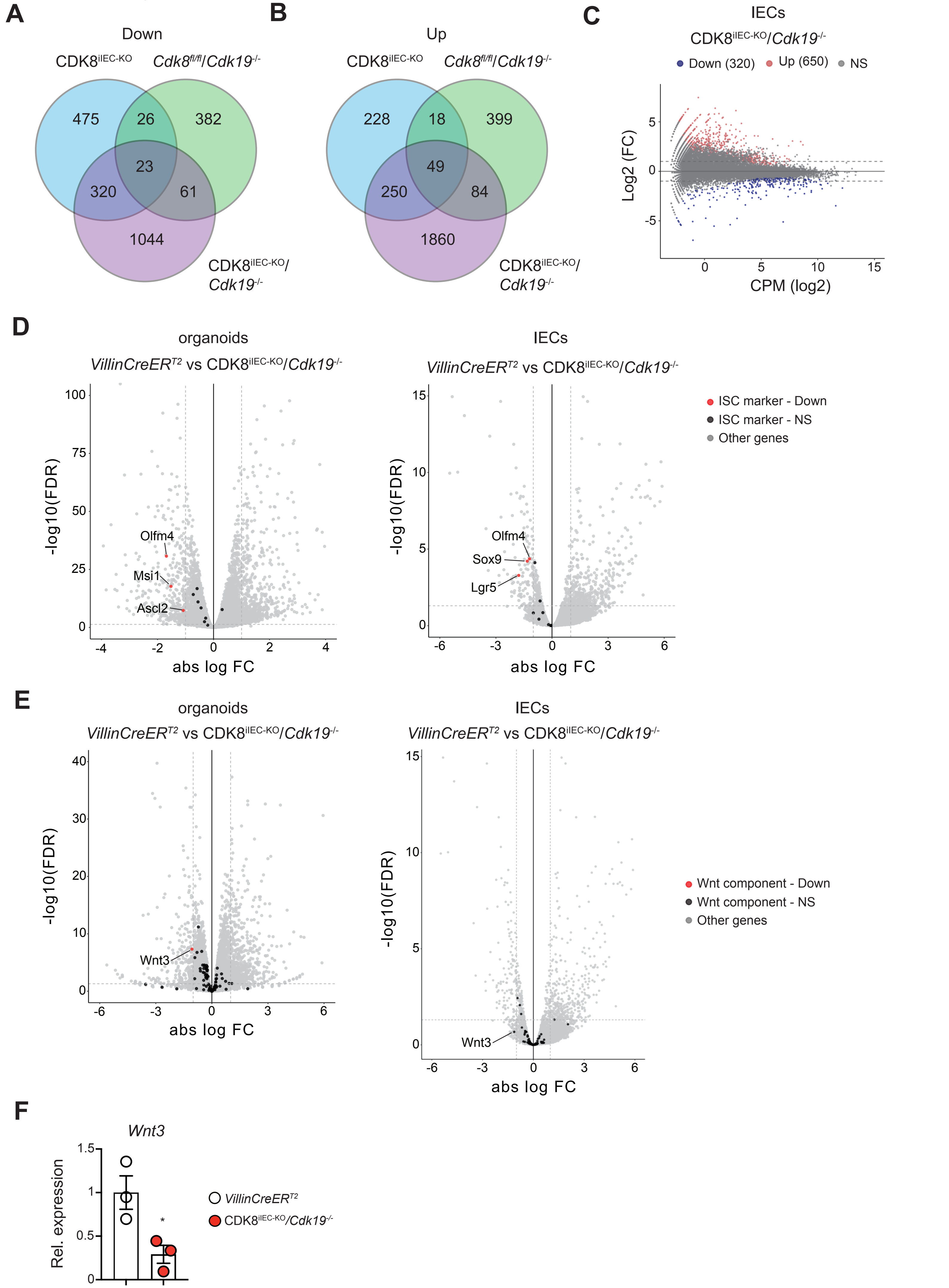

Figure S6. Transcriptomic analysis of the Mediator kinase-deficient intestinal epithelium.

(A) Venn diagram shows overlap of downregulated genes (FC <-1.5, FDR<0.05) from whole transcriptomic analysis (RNA-seq) in $CDK8^{iIEC\text{-}KO}$, $\mathit{Cdk8}^{fl/fl}$/$\mathit{Cdk19}^{-/-}$ and $CDK8^{iIEC\text{-}KO}$/$\mathit{Cdk19}^{-/-}$ intestinal organoids 7 days after 4-OHT treatment.

(B) Venn diagram depicts overlap of upregulated genes (FC >1.5, FDR<0.05) from whole transcriptomic analysis (RNA-seq) in $CDK8^{iIEC\text{-}KO}$, $\mathit{Cdk8}^{fl/fl}$/$\mathit{Cdk19}^{-/-}$ and $CDK8^{iIEC\text{-}KO}$ /$\mathit{Cdk19}^{-/-}$ intestinal organoids 7 days after 4-OHT treatment.

(C) MA-plots show differentially expressed genes from whole transcriptomic analysis (RNA-seq) in IECs from $CDK8^{iIEC\text{-}KO}$/$\mathit{Cdk19}^{-/-}$ mice compared to $\mathit{VillinCreER}^{T2}$ mice (14 days after first tamoxifen injection) as counts per million (CPM) (log2). Dashed lines mark log2 Fold Change (FC) cut-offs (log2FC>0.585, up and log2FC<-0.585, down).

(D) Volcano plots of whole transcriptome analyses (RNA-seq) in $CDK8^{iIEC\text{-}KO}$/$\mathit{Cdk19}^{-/-}$ IECs and organoids compared to $\mathit{VillinCreER}^{T2}$ IECs and organoids**.** The intestinal stem cell genes (n=11 total analyzed) significantly downregulated are named and marked in red. The remainder are marked black. Dashed lines mark log2 FC cut-offs (>1 (up), <-1 (down)) and -log10 FDR (>1.3, (FDR<0.05); significant).

(E) Volcano plots show whole transcriptome analyses (RNA-seq) in indicated comparison groups from IECs (14 days after 4-OHT treatment) and organoids (5 days after 4-OHT treatment)**.** Wnt pathway related genes (n=93 total) are marked. Wnt3 is named and marked in red. The remaining Wnt pathway related genes are marked black. Dashed lines mark log2 FC cut-offs (>1 (up), <-1 (down)) and -log10 FDR (>1.3, (FDR<0.05); significant).

(F) qRT/PCR analysis of Wnt3 gene expression in IECs from the same mouse groups in panel (**E)** above.

Data represents mean ± SEM. *P≤0.05, ** P≤0.01, ***P≤0.005, ****P≤0.001

Supplemental Figure 7

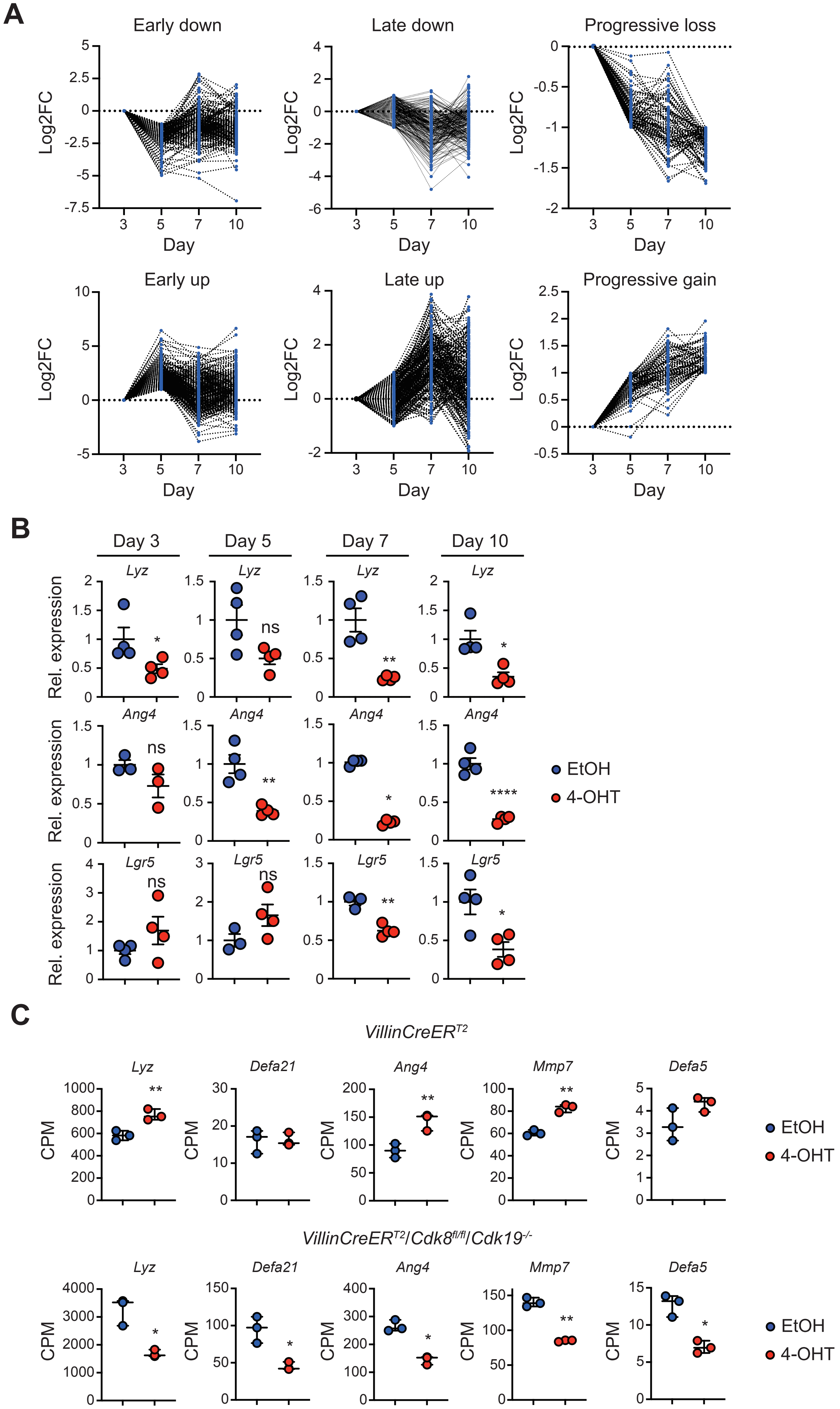

Figure S7. Temporal analysis of Mediator kinases regulated transcriptional programs.

(A) Cluster analysis of DEGs in *VillinCreER*^{T2}/*Cdk8*$^{fl/fl}$/*Cdk19*$^{-/-}$ intestinal organoids 3, 5, 7 and 10 days after EtOH and 4-OHT based on temporal expression patterns. Early down (n=326 genes), (Late down (n=196 genes), Progressive loss (n=89 genes), Early up (n=474 genes), Late up (n=261 genes), Progressive gain (n=83 genes).

(B) qRT-PCR for *Lyz*, *Ang4* and *Lgr5* in *VillinCreER*^{T2}/*Cdk8*$^{fl/fl}$/*Cdk19*$^{-/-}$ intestinal organoids 3, 5, 7 and 10 days after EtOH and 4-OHT treatment. Unpaired t-test and Mann-Whitney test used.

(C) Bar plots show gene expression (Counts Per Million, CPM) of indicated secretory cell type genes from the indicated mouse intestine organoid lines treated with 4-OHT or EtOH control. Unpaired t-test used

Data represents mean ± SEM. *$P \leq 0.05$, ** $P \leq 0.01$, ***$P \leq 0.005$, ****$P \leq 0.001$

Supplemental Figure 8

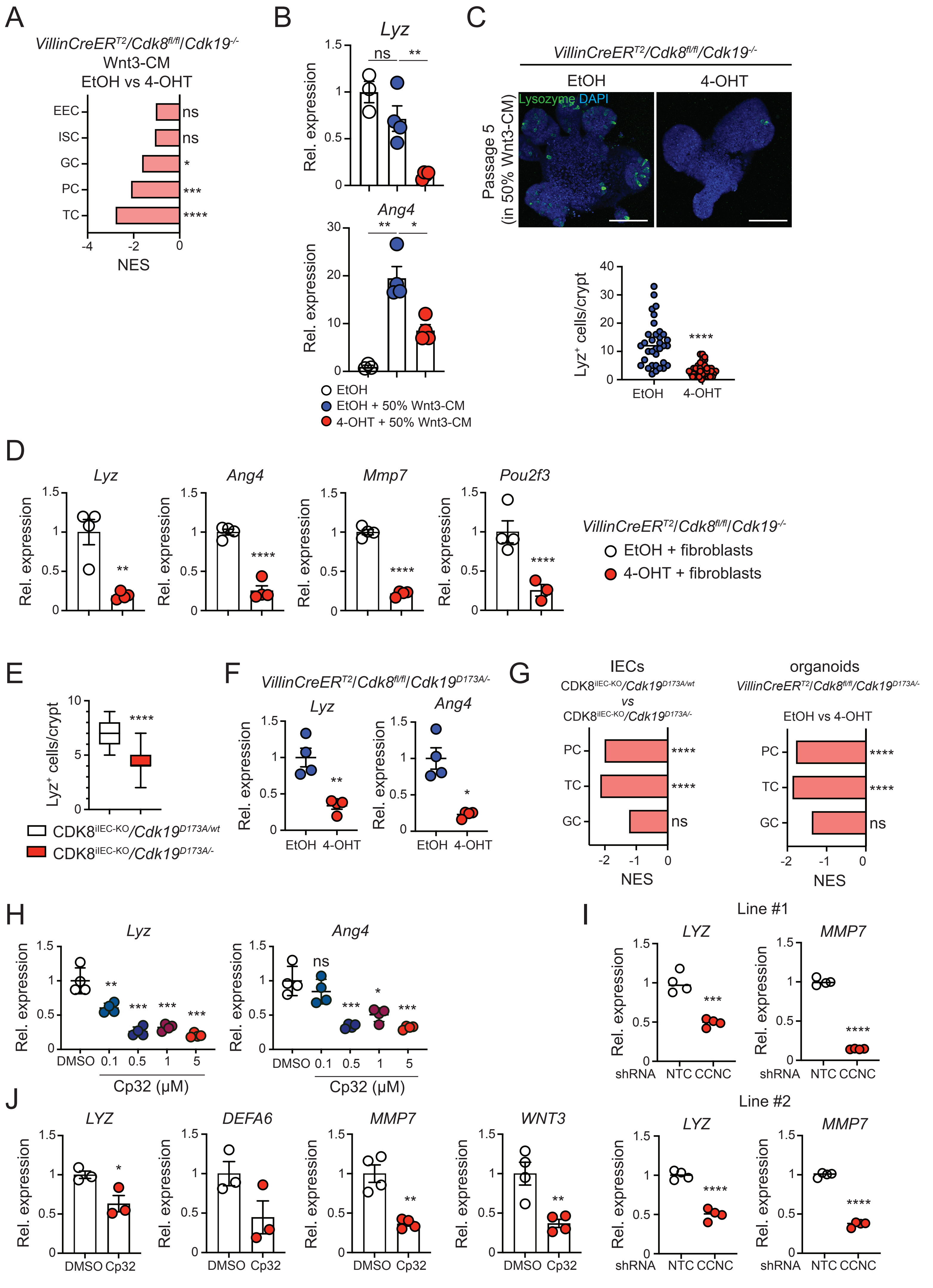

Figure S8. CDK8/19 kinase activity is required for intestinal epithelial secretory differentiation.

(A) GSEA for intestinal cell lineages showing Normalized enrichment score (NES) comparing EtOH and 4-OHT treated *VillinCreER^{T2}/Cdk8$^{fl/fl}$/Cdk19$^{-/-}$* organoids cultured in Wnt3-CM for 10 days. EEC, Enteroendocrine cells; ISC, Intestinal Stem cells; GC, Goblet cells; PC, Paneth cells; TC, Tuft cells.

(B) qRT-PCR analysis of *Lyz* and *Ang4* expression in *VillinCreER^{T2}/Cdk8$^{fl/fl}$/Cdk19$^{-/-}$* organoids after 5 passages in 50% Wnt3-CM. Unpaired t-test and Mann-Whitney test used.

(C) Representative merged images of Lysozyme (LYZ) (green) and DAPI (blue) immunofluorescence staining of intestinal organoids from the indicated conditions. Scale bar: 50 µm. Plot below shows quantification of Lyz+ cells (EtOH, n=34 crypts; 4-OHT, n=38 crypts). Mann-Whitney test used. Representative of two experiments shown.

(D) qRT-PCR gene expression analysis for the indicated secretory cells markers in EtOH- and 4-OHT treated *VillinCreER^{T2}/Cdk8$^{fl/fl}$/Cdk19$^{-/-}$* organoids co-cultured with primary intestinal fibroblasts for 19 days. Unpaired t-test was used.

(E) Quantification of LYZ IHC staining for CDK8$^{iIEC-KO}$/*Cdk19$^{D173A/wt}$* (mice n=4, villi n=136) and CDK8$^{iIEC-KO}$/*Cdk19$^{D173A/-}$* mice (mice, n=4; villi, n=149) as Box and whiskers (5-95 percentile). Unpaired t-test was used.

(F) qRT-PCR analysis of *Lyz* and *Ang4* expression in *VillinCreER^{T2}/Cdk8$^{fl/fl}$/Cdk19$^{D173A/-}$* organoids 7 days after EtOH or 4-OHT treatment. Unpaired t-test and Mann-Whitney test used.

(G) GSEA for intestinal secretory cell types showing Normalized Enrichment Score (NES) comparing the indicated genotypes and treatments in primary IECs and organoids. GC, Goblet cells; PC, Paneth cells; TC, Tuft cells.

(H) qRT-PCR analyses of *Lyz* and *Ang4* expression in *C57BL6* organoids after 7 days of treatment with indicated concentration of Cp32 or DMSO. Unpaired t-test and Mann-Whitney test used.

(I) qRT-PCR analyses of *LYZ* and *MMP7* expression in two independently derived human small intestinal organoid lines 96 hours after transduction with shRNAs targeting CCNC or non-targeting control (NTC). Unpaired t-test used.

(J) qRT-PCR for indicated secretory cell type genes in human small intestinal organoids after treatment with 0.5µM Cp32 or DMSO control for 7 days. Unpaired t-test used.

Data represents mean ± SEM. **P*≤0.05, ** *P*≤0.01, ****P*≤0.005, *****P*≤0.001

Supplemental Figure 9

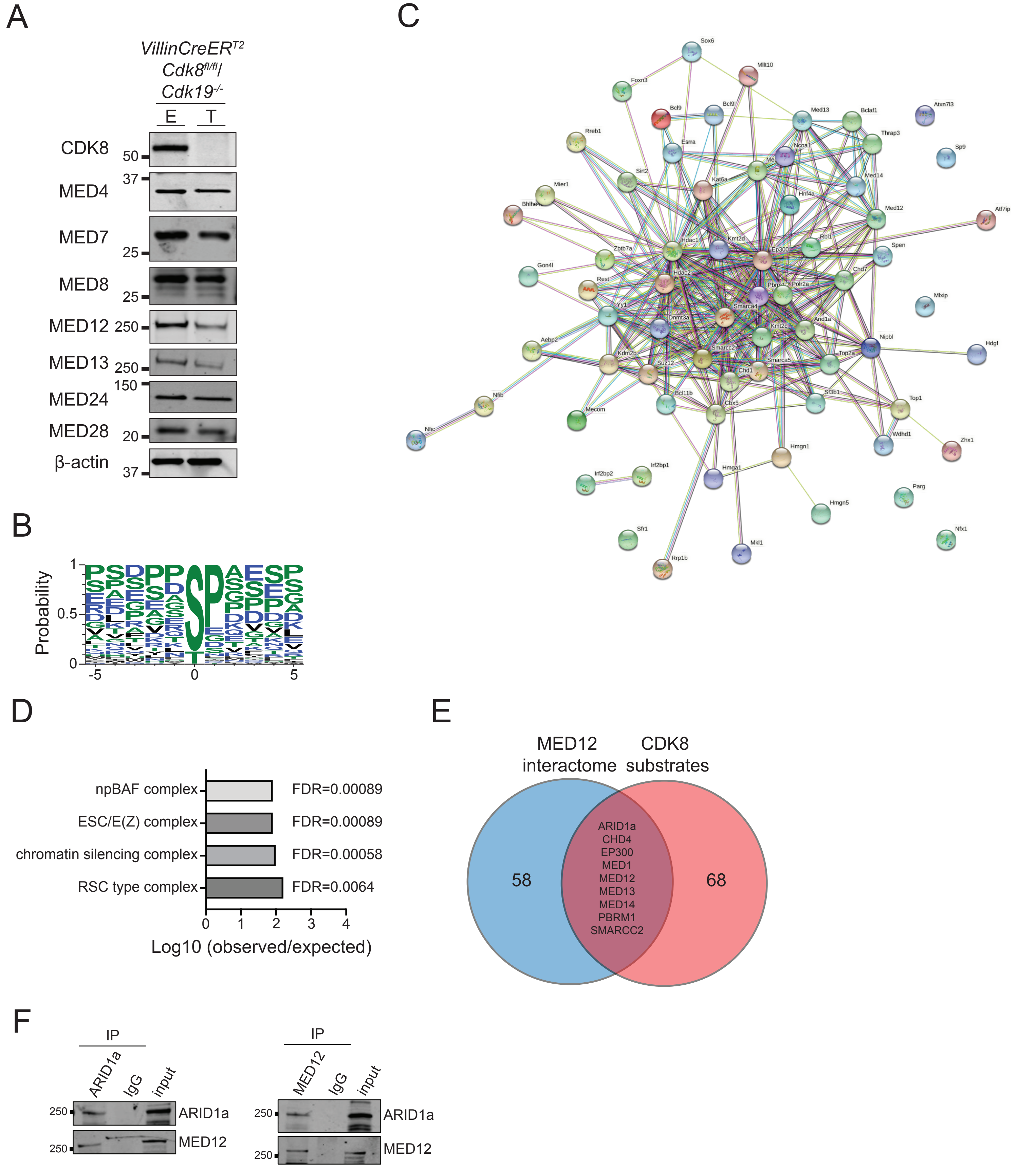

Figure S9. Phospho-proteomic analysis of the CDK8/19 interactome in intestinal epithelial cells.

(A) Immunoblot of protein lysates from *VillinCreER*^{T2}/*Cdk8*$^{fl/fl}$/*Cdk19*$^{-/-}$ intestinal organoids for Mediator components (7 days after treatment with EtOH and 4-OHT).

(B) Sequence logo plot represents 103 high confidence CDK8/19-associated phosphosites in chromatin related proteins. Amino acid sequence positions relative to the phosphorylation site (S/T) are shown in x-axis.

(C) STRING analysis of 68 identified high confidence substrates of CDK8/19.

(D) STRING analysis of the MED12 protein interaction network.

(E) Venn diagram showing overlap of MED12 interaction partners and chromatin associated CDK8/19 substrates.

(F) Co-immunoprecipitation for ARID1A and MED12 in primary human small intestinal epithelial cells. Isotype matched IgG protein was used as control.

Data represents mean ± SEM. **P*≤0.05, ** *P*≤0.01, ****P*≤0.005, *****P*≤0.001

Supplemental Figure 10

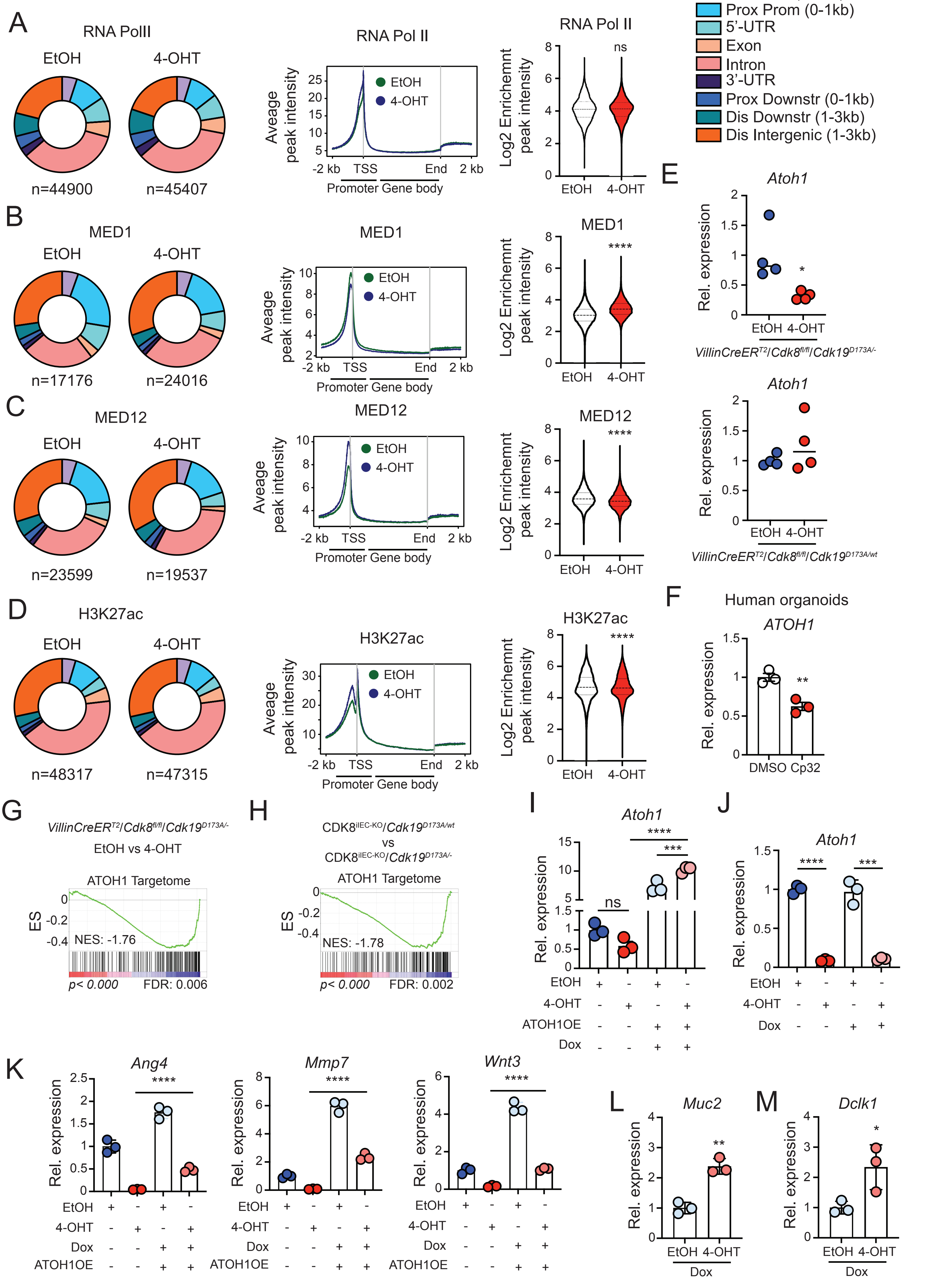

Figure S10. Genomic binding profiles define enhancer, Mediator, and transcriptional landscapes after CDK8/19 deletion.

(A) ChIP-seq signal analyses for RNA Pol II binding in *VillinCreER*^{T2}/*Cdk8*$^{fl/fl}$/*Cdk19*$^{-/-}$ intestinal organoids (treated with EtOH or 4-OHT for 7 days). (Left panel) Pie chart shows distribution of RNA Pol II across 9 pre-defined genomic regions (color coded as indicated in legend). Number of peaks is shown below the pie chart for each condition. (Middle panel) Composite plot shows average RNA Pol II binding at promoters and gene bodies in the indicated conditions. TSS, Transcriptional Start Site; End, end of gene body. (Right panel) Violin plot for RNA Pol II peak intensity in *VillinCreER*^{T2}/*Cdk8*$^{fl/fl}$/*Cdk19*$^{-/-}$ organoids 7 days after EtOH or 4-OHT treatment. Mann-Whitney test used.

(B-D) ChIP-seq signal analyses for **MED1 (B), MED12 (C), and H3K27AC (D)** in *VillinCreER*^{T2}/*Cdk8*$^{fl/fl}$/*Cdk19*$^{-/-}$ intestinal organoids (treated with EtOH or 4-OHT for 7 days) as described above in panel (**A**).

(E) qRT-PCR analysis of *Atoh1* expression in mouse intestinal organoids with the indicated genotypes 7 days after EtOH and 4-OHT treatment. Unpaired t-test used.

(F) qRT-PCR analysis of *ATOH1* expression in human small intestinal organoids after treatment with DMSO or 0.5µM Cp32 for seven days.

(G) GSEA for intestinal ATOH1 targetome in EtOH- and 4-OHT treated *VillinCreER*^{T2}/*Cdk8*$^{fl/fl}$/*Cdk19*$^{-/-}$ intestinal organoids 7 days after treatment.

(H) GSEA for intestinal ATOH1 targetome in primary IECs from mice with the indicated genotypes 14 days after tamoxifen injection.

(I) qRT-PCR analysis of *Atoh1* expression in *VillinCreER*^{T2}/*Cdk8*$^{fl/fl}$/*Cdk19*$^{-/-}$ intestinal organoids under the indicated conditions. *VillinCreER*^{T2}/*Cdk8*$^{fl/fl}$/*Cdk19*$^{-/-}$ organoids harbor a Dox-inducible Atoh1 overexpressing construct (ATOH1OE) as shown. RNA was harvested for qRT-PCR 7 days post EtOH or 4-OHT treatment and 3 days after Dox treatment to induce ATOH1 expression (24hrs with Dox, followed by 48hrs with no Dox). One-way ANOVA with Tukey's multiple comparisons test. Representative data from three independent experiments shown.

(J) qRT-PCR analysis of *Atoh1* expression in parental *VillinCreER*^{T2}/*Cdk8*$^{fl/fl}$/*Cdk19*$^{-/-}$ organoids treated with the indicated conditions and as described in panel **(I)** above. One-way ANOVA with Tukey's multiple comparisons test. Representative data from three independent experiments shown.

(K) qRT-PCR analysis of the indicated genes using *VillinCreER*^{T2}/*Cdk8*$^{fl/fl}$/*Cdk19*$^{-/-}$ organoids in the presence and absence of exogenous ATOH1 expression, as described in panel **(I)** above. One-way ANOVA with Tukey's multiple comparisons test. Representative data from three independent experiments shown.

(L, M) qRT-PCR analysis of *Muc2* **(L)** and *Dclk1* **(M)** expression in ATOH1-overexpressing *VillinCreER*^{T2}/*Cdk8*$^{fl/fl}$/*Cdk19*$^{-/-}$ organoids under the indicated conditions.

Data represents mean ± SEM. *P≤0.05, ** P≤0.01, ***P≤0.005, ****P≤0.001

Supplemental Figure 11

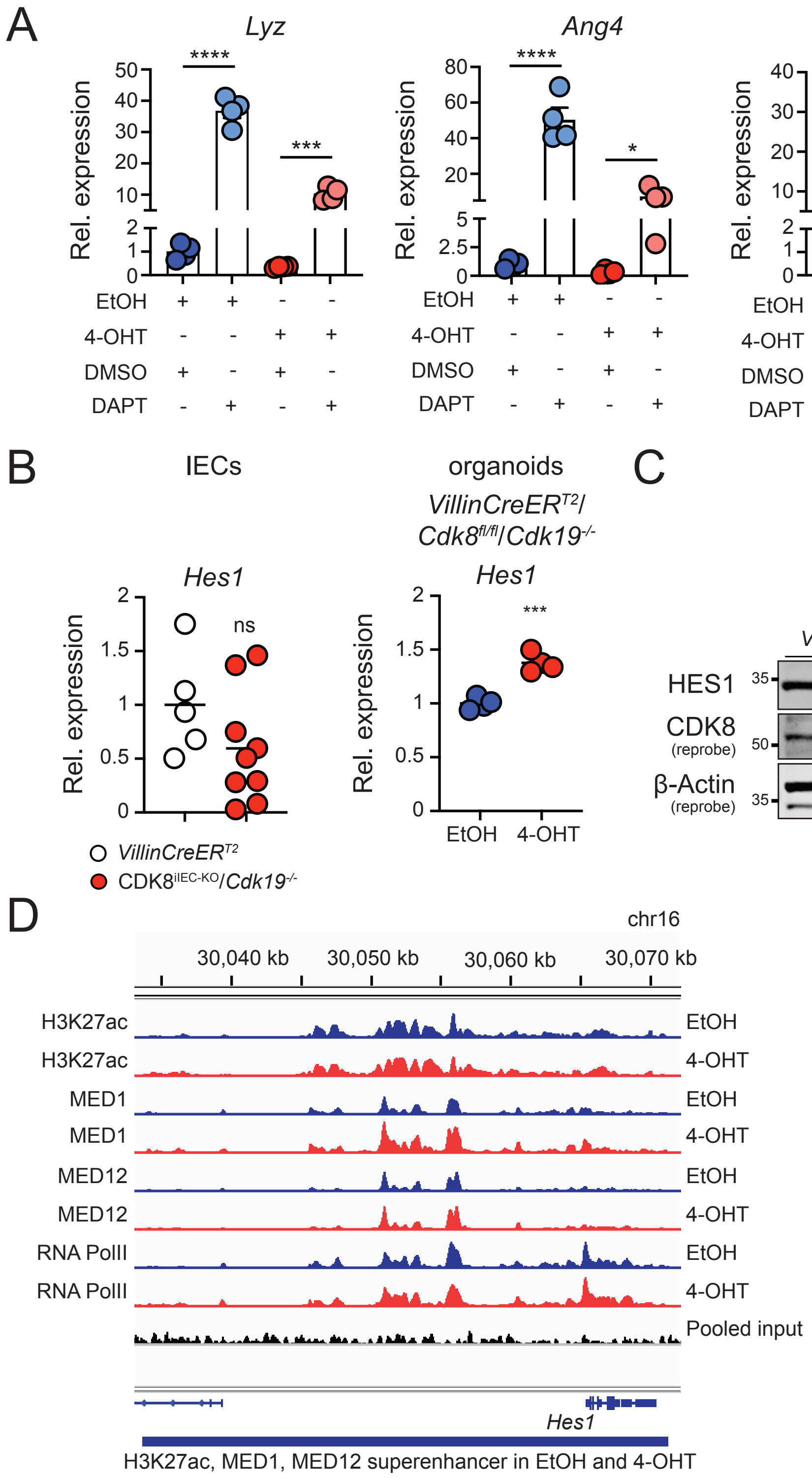

Figure S11. CDK8/19 regulate Atoh1 transcription in a Notch-independent manner.
(A) qRT-PCR analysis of *Lyz, Ang4* and *Mmp7* expression in EtOH and 4-OHT treated *VillinCreER^{T2}/Cdk8$^{fl/fl}$/Cdk19$^{-/-}$* organoids after 48hr treatment with DMSO or DAPT. Unpaired t-test and one-way ANOVA with Tukey's multiple comparisons test were used.
(B) qRT-PCR analysis for *Hes1* gene expression in *VillinCreER^{T2}/Cdk8$^{fl/fl}$/Cdk19$^{-/-}$* intestinal organoids (7 days after EtOH and 4-OHT treatment) and in SI IECs from *the* indicated mouse groups (2 weeks post tamoxifen injections). Unpaired t-test was used.
(C) Immunoblots show HES1, CDK8, CDK19 protein levels in organoids and IECs from the indicated genotypes. b-Actin is shown as a loading control.
(D) Genome browser track for H3K27ac, MED1, MED12 and RNA Pol II ChIP-seq signals neighboring the *Hes1* locus in *VillinCreER^{T2}/Cdk8$^{fl/fl}$/Cdk19$^{-/-}$* organoids 7 days after EtOH or 4-OHT treatment. Pooled input is shown as a control.
Data represents mean ± SEM. *$P \leq 0.05$, ** $P \leq 0.01$, ***$P \leq 0.005$, ****$P \leq 0.001$

SUPPLEMENTAL METHODS

Mice

*Cdk8^fl/fl^* (1), *Cdk19^-/-^* (obtained from JAX Lab) and *VillinCreER^T2^* (2) mice have been described previously. *C57BL/6J* mice were obtained from the Monash Animal Research Platform. The *Cdk19^D173A^* knock-in mice were generated by the Melbourne Advanced Genome Editing Centre (MAGEC) at Walter and Eliza Hall Institute (WEHI) on a *C57BL/6J* background. The oligo donor (cctgcatgactctgatcatatggcaaaatgagttttatatatttgttttatttttcagCTGCGATGGGTTTTGCCA GATTATTCAATTCTCCCCTAAAGCCACTCGCAGATTTGGATCCAGTGGTTGTGA CATTTTGG) (40 ng/µl^{-1}) alongside 20 ng/µl^{-1} Cas9 mRNA, 10 ng/µl^{-1} sgRNA were injected into the cytoplasm of fertilized *C57BL/6J* one-stage embryos. One day later, two-cell stage embryos were transferred into pseudo-pregnant females, and viable offspring were genotyped by sequencing. Properly targeted mice were backcrossed for at least 5 generations before being used to generate crossings described in the main text.

All mice were maintained at Monash Medical Centre (MMC) and Monash University SPF animal facilities. Mice were kept in a 12 h light cycle and given a chow diet *ad libitum*. Females with the indicated genotypes between 8 to 12 weeks of age were assigned to groups at random. VillinCreERT2 recombinase was induced by an intraperitoneal injection of 80 mg/kg tamoxifen every 24 h for 5 consecutive days. Littermates not carrying the VillinCreERT2 transgene and mice VillinCreERT2 transgene-only expressing mice were used as controls in all experiments. Genotypes of individual mice were confirmed using tail DNA after tissue collection.

IEC isolation

Small intestines were cut open longitudinally and washed in ice-cold PBS to remove luminal content. Intestines were incubated in 1 mM DTT, 2 mM EDTA (diluted in 150 mM NaCl, 2.7 mM KCl, 1.2 mM KH_2PO_4, 680 mM NaH_2PO_4) for 30 minutes on ice. To release IECs, intestines were vortexed for 2 minutes and the intestines removed. The supernatant was centrifuged, resuspended in cold PBS, transferred to Eppendorf tubes and centrifuged again at full speed. The supernatant was aspirated, the IEC pellet either directly used for downstream application or snap frozen on dry ice and stored at -80°C until further use.

Crypt isolation and culturing of intestinal organoids

Small intestinal organoids were isolated and cultured as previously described with few alterations (3). Instead of recombinant R-Spondin and Noggin, conditioned media was used. R-Spondin conditioned media was obtained from Helen Abud's lab (Monash University, Australia) and added to culturing media at a final concentration of 5% (v/v). Noggin-conditioned media and Wnt3a conditioned media were produced in-house and used at a final concentration of 10% and 50% (v/v) respectively. Organoids were maintained in 24-well plates (Corning) and culturing media was changed every 3-4 days, and during Cp32 experiments, every other day. To induce Cre-recombinase activity, 100 nM 4-hydroxytamoxifen (4-OHT) was added for 24 h and the medium exchanged after. EtOH-treated organoids were used as a vehicle control in every experiment.

Human organoid cultures

Duodenum tissues were collected for organoid isolation and transferred into ice-cold PBS until use. Using scissors, the stroma was removed, and epithelium cut into small pieces of 1-2 mm^3 size. Intestinal fragments were washed in PBS and incubated in 2mM EDTA at 4°C for one hour. Crypts were washed in 10% FBS in PBS and filtered through a 70 µm strainer. After centrifugation, crypts were resuspended in 50 µl Matrigel, plated out onto 24-well plates and overlayed with 500 µl Stemcell Technologies Intesticult™ Human Organoid Growth Media (OGM). Intesticult OGM is especially formulated to enrich for stem- and progenitor cells. For improved multi-differentiation capacity preserving *in vivo* cellular diversity, organoids were cultured in a refined media in all experiments as recently reported (4).

Organoid growth assays

Organoid growth assays were performed in 48-well plates. Organoids with the indicated genotype were counted manually and seeded out at uniform density of 50-80 organoids per well. At the start and end of each passage, organoid viability was assessed by Presto Blue (A13262, Thermo Fisher) or Alamar Blue HS (A50101, Thermo Fisher) according to manufacturer's instructions. Incubation time was 2 h and matrigel-only wells were used as a baseline. After measurements, organoids were

washed and culture media was added. At the end of each passage (5-7 days), organoids were reseeded in a 1:4 split ratio.

Generation of lentiviral constructs and organoid transduction

To generate lentivirus, the plasmid of interest in combination with psPAX2 (packaging plasmid) and Pmd2.g (envelope plasmid) were transfected into HEK293T cells. High-titer lentiviral particles were produced with Lenti-XTM Concentrator (Takara).

To establish organoids with doxycycline inducible Atoh1 expression, an Atoh1-pcw57.1 plasmid was generated by a LR recombination reaction using Clonase (Thermo Fisher, 11791020) between Twist pENTR containing the mouse Atoh1 cDNA sequence (pTwist, custom design) and pcw57.1 plasmid. For CRISPRi and CRISPRa screening, nuclease dead Cas9 fused to KRAB transcriptional repressor (dcas9-KRAB, Addgene #110820) and transcriptional activator VP64 (dcas9-VP64, Addgene #61425) plasmids were transduced into C57/BL6 and *VillinCreER*^{T2}/*Cdk8*$^{fl/fl}$/*Cdk19*$^{-/-}$ organoids respectively. Generated ncCas9-KRAB and ncCas9-VP64 expressing organoid lines were selected with blasticidin (8µg/ml) for at least one week prior to experiment and maintained in selection for the whole duration of the experiments. To activate or repress expression of Atoh1-associated SE, sgRNAs with NGG PAM were designed to target *Atoh1* promoter and four individual peaks within the *Atoh1* super enhancer. Oligonucleotides pairs for each gRNA were then synthesized and annealed to LentiGuide-Puro (Addgene #52963) for CRISPRi or pXPR_502 (Addgene #96923) for CRISPRa, both beforehand digested with Esp3I (ER0451, Thermo). sgRNA used in this study are listed in Table S7. Puromycin (1.5ug/ml) was added into culture media to select clones with integrated constructs. Plasmid for shRNA-mediated targeting of human CCNC and control were purchased from Merck (TRCN0000077830).

Prior to transduction, organoids were dissociated with a 27G syringe and Tryple, plated as single cell suspension with 50 µM Y-27632, 10 mM CHIR-99021 and 100 nM nicotinamide. Spinoculation (600 g, 60 min, 32°C) was performed to enhance transduction efficiency. Organoid-virus mixture was incubated for 4 hours before replating. After 24h, puromycin (1.5µg/ml) was added into culture media to select clones with integrated constructs. Puromycin-resistant organoids were maintained in selection for the whole duration of the experiments.

Intestinal fibroblast isolation and culture

The small intestine was harvested, cut open longitudinally and washed with ice-cold PBS. The tissue was transferred into a 50 ml Falcon tube with 10 ml PBS + 1 mM DTT and incubated for 10 min at 37°C on a shaker. Tissue was then collected, washed shortly with PBS and incubated with 10 ml PBS+ 1.5 mM EDTA for 15 min at 37°C. Afterwards small intestines were washed with PBS (~10 ml) at least 3 times by vigorously shaking the tube until the supernatant was clear. The tissue was placed in a Petri dish and cut into small pieces (1-5 mm) with scissors. In order to isolate fibroblasts, cut tissue pieces were resuspended with 5-10 ml pre-warmed Minimum Essential Eagle Medium supplemented by glutamine (1%), penicillin (2%), Collagenase A (1-1.5 mg/ml) and Dispase (1-1.5 mg/ml) and digested at 37℃ for 35 to 60 min. The supernatant was collected and passed through a 70µm filter and then centrifuged for 10min at 280 g. The pellets were resuspended in RPMI-1640 with 10% FBS and 2% P/S and seeded out into a culturing plate. After 24 hours, the media was replaced.

Histology

Intestinal sections were fixed in 10% normal buffered formalin, embedded in paraffin and cut in 4 µm sections. H&E staining and Periodic acid-Schiff (PAS) reaction was performed according to standard protocols. Endogenous alkaline phosphatase activity was visualised with Alkaline Phosphatase Substrate Kit (SK-5100, Vector Laboratories) following manufacturer's instructions. Sections were rehydrated and antigen retrieval was done using citrate buffer or proteinase K treatment. The following primary antibodies, diluted at 1:1000 in 10% normal goat serum were used: Olfm4 (39141S, CST), Ki67 (ab16667, Abcam), Dclk1 (62257S, CST), Lysozyme (EC 3.2.1.17, Agilent). Biotinylated secondary antibody (BA-1000-1.5, Vector Laboratories) was used in combination with Vectastain Elite ABC system (PK-6100, Vector Laboratories), and staining was visualised with ABC Kit Vectastain Elite (Vector Laboratories) and DAB substrate. Incubation time with DAB substrates was equal for all samples. All sections were counterstained with Haematoxylin.

Immunofluorescence staining of organoids

For immunofluorescent imaging, organoids with indicated genotypes were cultured in imaging chambers (Sarstedt). To enable better penetration of the antibody, organoids

were seeded out in a 1:1 matrigel media mixture. Organoids were fixed with 4% paraformaldehyde at room temperature for 1 h and blocked in PBS with 0.5% Triton-X 100, 10% NGS for 30 min at room temperature. Organoids were incubated with a primary antibody against Lysozyme (1:200, EC 3.2.1.17, Agilent) diluted in blocking buffer with 0.1% Tween-20 and overnight at 4℃. Slides were washed with PBS followed by secondary antibody (1:500, A-11008, ThermoFisher) incubation at room temperature for 1 hour. DAPI (1:10,000) was used to visualise cell nuclei. Confocal images were captured on a Nikon C1 inverted confocal microscope.

Immunoblotting

Protein extracts from SI IECs and organoids were prepared using high salt RIPA buffer (20 mM HEPES, pH 7.6; 350 mM NaCl; 20% glycerol; 1 mM MgCl2; 0.5 mM EGTA; 0.1 mM EGTA; 1% NP-40) supplemented with 0.05 M NaF, 20 mM sodium orthovanadate, 1mM PMSF and 1x protease inhibitor cocktail (Sigma, P2714-1BTL) to inhibit protease activity. Cell lysates were separated on SDS-PAGE and transferred to Immobilon-FL PVDF membranes (Millipore) and analyzed by immunoblotting. Membranes were probed with primary antibodies against the following proteins: CDK8 (A302-5-1A, Bethyl Laboratories), CDK19 (HPA007053, Merck), HES1 (2922-1, Epitomics), P-STAT1 (#8826, CST), STAT1 (#9172, CST), MED1 (A300-793A, Bethyl Laboratories), MED12 (A300-774A, Bethyl Laboratories), ARID1A (sc32761, Santa Cruz), MED4 (ab129170, Abcam), MED7 (ab187146, Abcam), MED8 (sc-365960, Santa Cruz), MED13 (301-278A, Bethyl Laboratories), MED24 (A301-472A, Bethyl Laboratories) and MED28 (HPA035900, Sigma-Aldrich). Membranes were incubated with anti-rabbit secondary antibodies coupled to IRDye680 (926-68071, Licor) or IRDye800 (926-32211, Licor) at 1 µg/ml for one hour and visualised using Odyssey CLx. DyLight800 labelled Beta-Actin (MA5-15739-D800, ThermoFisher) was used as a loading control.

Gene expression analysis

Total RNA was extracted using RNAzol RT (Sigma Aldrich), TRIzol™ (ThermoFisher) or RNeasy Columns (Qiagen) according to manufacturer's instructions. For RNA-sequencing experiments, RNA was extracted with Qiagen RNeasy columns. To extract RNA from IECs, first a phenol-chloroform extraction was performed and RNA was purified on RNeasy Columns (Qiagen) according to standard protocols. An on-

column DNA digest was performed. Depending on the experiment, RNA was used for RT-PCRs directly using GoTaq® 1-Step RT-qPCR system (A6020, Promega) or cDNA was synthesized using SuperScript™ III First Strand (#18080-051, invitrogen) or RevertAid First Strand cDNA Synthesis Kit (#K1622, Thermofisher). Gene expression analysis was performed using SYBR Green and Taqman expression with a total reaction volume of 10 µl. Rplp was used as a reference gene in all experiments and data was analysed according to the ΔΔCT method. Sequences of Taqman probes and SYBR primers used in this study are shown in Table S7.

RNA-sequencing

For all RNA-sequencing experiments, RNA was extracted with Qiagen RNeasy columns according to manufacturer's instructions including on column DNase treatment. All RNAseq samples were submitted for sequencing in biological triplicates. The library preparations and RNAseq were performed at Beijing Genomics Institute (Hong Kong, China) with a minimum of 20 million read counts per sample using paired-end 100bp sequencing. Briefly, ribosomal RNA was depleted from the total RNA samples. To construct the library, RNA samples underwent random fragmentation, reverse transcription, adaptor ligation, PCR amplification, single strand separation and DNA nanoball synthesis. The library was then sequenced on a DNBSEQ G-400 platform. Data processing was automated using the RNAsik pipeline. This analysis included read quality assessment by FastQC analysis (version 0.11.5), mapping to the mouse genome (GRCm38) by the STAR aligner (5) and gene-level reads quantification using the featureCounts function of the Rsubread package (6). Differential expression analysis was performed using the Degust platform (Powell, n.d.) with the EdgeR method (7) and IDEP.91 platform using the DESeq2 method (8). A permissive threshold was used to define significant differential expression of genes in the experimental groups compared to the control (absolute log fold change (absFC) ≥ 0.585, false discovery rate (FDR) ≤ 0.05). Gene Set Enrichment Analysis (GSEA) was used to examine significantly enriched gene sets and the associated annotated molecular signatures. Specifically, the GSEA preranked method was used with a "classic" enrichment statistic based on the log2FC values determined in the differential expression analysis.

Chromatin extraction and ChIP reactions

Samples were sent to Active Motif for ChIP-Seq. Active Motif prepared chromatin, performed ChIP reactions, generated libraries, sequenced the libraries and performed basic data analysis. In brief, cells were fixed with 1% formaldehyde for 15 min and quenched with 0.125 M glycine. Chromatin was isolated by adding a lysis buffer, followed by disruption with a Dounce homogenizer. Lysates were sonicated and the DNA sheared to an average length of 300-500 bp with Active Motif's EpiShear probe sonicator (53051) and cooled sonication platform (53080). Genomic DNA (Input) was prepared by treating aliquots of chromatin with RNase, proteinase K and heat for de-crosslinking, followed by SPRI beads clean up. Eluted DNA was quantified by Clariostar. Extrapolation to the original chromatin volume allowed quantification of the total chromatin yield. An aliquot of chromatin (25 ug) was precleared with protein A agarose beads (Invitrogen). Genomic DNA regions of interest were isolated using 6 µl of MED12 (A300-774A, Bethyl Laboratories), 6 µl of MED1 (A300-793A, Bethyl Laboratories), 5 µl of Total RNA Polymerase II (91151, Active Motif), 4ul of H3K27Ac (39133, Active Motif) and 6 µl of ARID1A (NB100-55334, Novus) antibodies. Complexes were washed, eluted from the beads with SDS buffer, and subjected to RNase and proteinase K treatment. Crosslinks were reversed by incubation overnight at 65 C, and ChIP DNA was purified by phenol-chloroform extraction and ethanol precipitation. Quantitative PCR (QPCR) reactions were carried out in triplicate on specific genomic regions using SYBR Green Supermix (Bio-Rad). The resulting signals were normalized for primer efficiency by carrying out QPCR for each primer pair using Input DNA.

ChIP Sequencing

Illumina sequencing libraries were prepared from the ChIP and Input DNAs using the standard consecutive enzymatic steps of end-polishing, dA-addition, and adaptor ligation using Active Motif's custom liquid handling robotics pipeline. After the final PCR amplification step, the resulting DNA libraries were quantified and sequenced on Illumina NexSeq 500. Sequences (75 bp, single end) were aligned to the mouse genome (mm10) using the BWA algorithm (default settings) (9). Duplicate reads were removed and only uniquely mapped reads (mapping quality >= 25) were used for further analysis. Alignments were extended *in silico* at their 3'-ends to a length of 200 bp, which is the average genomic fragment length in the size-selected library and

assigned to 32-nt bins along the genome. The resulting histograms (genomic “signal maps”) were stored in BigWig files. Peaks were identified using the MACS 2.1.0 algorithm at a cutoff of p-value = 1e-7 (10). Peaks that were on the ENCODE blacklist of known false ChIP-Seq peaks were removed. Signal maps and peak locations were used as input data to Active Motif's proprietary analysis program, which creates Excel tables containing detailed information on sample comparison, peak metrics, peak locations and gene annotations. Galaxy was used to generate bed files. GREAT algorithm (version 4.0.4) was used to define cis regions within -5Kb and + 1Kb from TSS (11). IGV was used to generate peak intensity plots. Intervene was used to perform overlapping analysis between the genomic regions (12).

ChIP-qPCRs

Chromatin immunoprecipitation was performed using ChIP-IT® Express Enzymatic Magnetic ChIP-Kit (53009, 53035, Active Motif) according to manufacturer’s instructions. For immunoprecipitation, 2µg ARID1A antibody (sc32761, Santa Cruz) and 2 µg MED12 antibody (A300-774, Bethyl Laboratories) were used. The primers used for ChIP-RT-PCR are listed in Table S7.

Whole cell proteomics and phospho-proteomics

Organoid pellets were lysed in 4% sodium deoxycholate (SDC) at 95°C for 5 min. Following sonication, protein concentration was determined using BCA protein assay (Thermo Fisher Scientific) and 700 µg of lysates was used as starting material. Reduction and alkylation were performed at 95°C for 5 min using 10 mM TCEP and 40 mM 2-chloroacetamide (CAM) at pH 7-8. Lysates were digested with LysC and Trypsin at an enzyme-to-substrate ratio of 1:100 and incubated overnight at 37˚C with shaking at 1500 rpm.

For total proteomics, 50 ug of the digest protein lysate were cleaned through C18 columns and eluted in 50%ACN/0.1% TFA for mass spectrometry analysis. The rest of the lysates were subjected to phosphoproteomics enrichment using TiO2 as previously described (13). In brief, SDC was precipitated and removed by the addition of 400 µl isopropanol and 100 µl of 48% TFA/8 mM KH2PO4 solution. The samples were centrifuged at 2000 g for 10 min and supernatant collected. 5 mg of TiO_2beads were resuspended in 6% TFA/80% ACN (v/v) prior to addition to each sample. The

samples were incubated at 40°C with shaking at 2000rpm for 5 min. The beads were then washed four times with 1 ml 5% TFA/60% ISO (v/v), loaded onto C8 StageTips and the phosphopeptides eluted with 60 µl 5% ammonia solution/40% acetonitrile (ACN) (v/v). The eluates were dried in a vacuum concentrator to a volume of ~15 µl. The dried down samples were resuspended in 1% TFA in isopropanol (ISO) (v/v), transferred onto SDB-RPS StageTips and centrifuges to dryness at 1500 g. The SDB-RPS StageTips containing the phosphopeptides were subsequently washed with 1% TFA in ISO (v/v) followed by 0.2% TFA/5% ACN (v/v). The phosphopeptides were eluted with 0.1% ammonia solution/60% ACN (v/v) and dried to completeness in a vacuum concentrator. Samples were resuspended in 6µl 0.1% FA/2% ACN (v/v) for mass spectrometry analysis.

Samples were analyzed on an UltiMate 3000 RSLC nano LC system (Thermo Fisher Scientific) coupled to an Q Exactive HF Mass Spectrometer (Thermo Fisher Scientific). The samples were injected onto a 100 µm, 2 cm nanoviper Pepmap100 trap column, eluted and separation performed on a RSLC nano column 75 µm x 50 cm, Pepmap100 C18 analytical column (Thermo Fisher Scientific). Peptides were eluted using an LC gradient of 2.5 - 42.5% ACN/0.1%FA at 250 nL/min over 150 min. The mass spectrometer was operated in the DDA mode to automatically switch between full MS scans and subsequent MS/MS acquisitions. Survey full scan MS spectra (m/z 300–1750) were acquired in the Orbitrap with 70,000 resolution (at m/z 200) after accumulation of ions to a 1 x 10^6 target value with a maximum injection time of 30 ms. Dynamic exclusion was set to 30 sec. Up to 10 most intense charged ions ($z \geq +2$) were sequentially isolated and fragmented in the collision cell by higher-energy collisional dissociation (HCD) with a fixed injection time of 120 ms, 17,500 resolution and automatic gain control (AGC) target of 1 x 10^5.

The raw MS files were processed using MaxQuant software (v6.12.0) with the following parameters: FDR <0.01, precursor mass tolerance set to 20 ppm, fragment mass tolerance set to 0.5 Da, minimum peptide length of six amino acids, enzyme specificity set to Trypsin/P & LysC, mouse uniport database (v2020) and a maximum number of missed cleavages of 2. Fixed modifications were limited to Carbamidomethyl (C) and variable modifications were set to Oxidation (M), Acetyl (protein N-term), and Phospho (14). The ‘match between runs’ option in MaxQuant

was selected using the default parameters. The fold change values were calculated from triplicate independent experiments using the limma package in R.

Immunoprecipitation

Organoids were lysed in NTEN lysis buffer (250 mM NaCl, 5mM EDTA, 50 mM HEPES, 0.5% NP-40) freshly supplemented with Halt™ Protease and Phosphatase Inhibitor Cocktail (78441, Thermo Fisher Scientific) and protein concentration was determined with Pierce BSA Protein Assay Kit (23225, Thermo Fisher Scientific). Either 500 µg or 1 mg of protein was used for IP with 1 µg of MED1 (A700-037, Bethyl Laboratories), MED12 (A300-774, Bethyl Laboratories), ARID1A (sc32761, Santa Cruz) or rabbit and mouse IgG isotype controls (31235 and 31903, Thermo Fisher Scientific) and incubated overnight at 4°C on a rotating wheel. Dynabeads Protein A (for rabbit AB) and G (for mouse AB) (Thermo Fisher) were washed twice in the NTEN buffer and incubated with antibody-lysates for four hours at 4°C on a rotating wheel. The samples were washed five times in NTEN lysis buffer before proceeding with isobaric labelling or elution from Dynabeads with 1x Laemmli buffer.

Isobaric labelling and quantitative proteomics

Buffer conditions for IPs subjected to isobaric labelling and quantitative proteomics were changed by washing twice with 100 mM TEAB buffer before following TMT™ sixplex Isobaric Mass Tagging Kit (90064, Thermo Fisher Scientific) manufacturer's instructions. Acetone precipitation was omitted and trypsin digested peptides were separated from beads immediately before peptide labelling. Labelled peptides were purified using Pierce™ C18 Spin columns (89870, Thermo Fisher Scientific) and dried in a Speed Vac. LC-MS/MS Analysis Samples were analyzed using an Orbitrap Exploris 480 mass spectrometer (Thermo Scientific) coupled with an Easy-nLC 1200 (Thermo Scientific), Nanospray Flex ion source (Thermo Scientific), and column oven heater (Sonation). One µL of the sample was directly loaded onto the column. Peptides were eluted with a linear gradient (EasyNanoLC 1200, Thermo Fisher Scientific) ranging from 6%–30% Solvent B (0.1%FA in 90% ACN) over 84 min, 30%–90% B over 9 min and held at 90% B for 5 min at 200 nl/min. MS/MS were acquired on a Thermo Orbitrap Exploris 480 (Thermo Fisher Scientific) in data-dependent acquisition. LC-MS/MS data were analyzed using Peaks X plus (BSI) database search against mouse proteome (SwissProt) with the following search parameters: Parent

mass error tolerance for parent and fragment mass was set to 20 ppm and 0.02 Da respectively; digestion mode was set to tryptic, with TMT- 6plex and carbamidomethylation (C) as fixed modification and Oxidation(M), Deamidation (N,Q)Acetylation (N-term and K), Phosphorylation (S) as variable modifications (a maximum of 2 per sequence) with False Discovery Rate (FDR) of 1%. To enable assignment of MS2 reporter ion data to respective samples, PeaksQ. quantitation was used with a tight quantification mass tolerance of 5.0 ppm utilising peptides that matched the FDR threshold of 1%. Hits were filtered for Log2FC <-1 and a p-value of 0.05 and further filtered for nuclear proteins.

scRNA-seq dataset analysis

Intestinal single cell 3' gene expression data for mouse (15) and human (16) were downloaded. Gene expression was normalised using the formula log2 (counts per thousand + 1). Owing to the low read depth of single cell RNA sequencing, the *ALRA* algorithm was used to impute the expression of genes (17). Owing to its large size, the Elmentaite dataset was subsetted to include only Tuft cells, TA cells, stem cells, Paneth cells, Goblet cells and Enterocytes. These cells were then simple-randomised into 1 of 20 sub-datasets, and each sub-dataset was processed by the *ALRA* algorithm separately. Violin and dot plots were generated using the *Seurat* package (18).

References

1. McCleland ML, Soukup TM, Liu SD, Esensten JH, de Sousa e Melo F, Yaylaoglu M, et al. Cdk8 deletion in the Apc(Min) murine tumour model represses EZH2 activity and accelerates tumourigenesis. *J Pathol.* 2015;237(4):508-19.
2. el Marjou F, Janssen KP, Chang BH, Li M, Hindie V, Chan L, et al. Tissue-specific and inducible Cre-mediated recombination in the gut epithelium. *Genesis.* 2004;39(3):186-93.
3. Sato T, Vries RG, Snippert HJ, van de Wetering M, Barker N, Stange DE, et al. Single Lgr5 stem cells build crypt-villus structures in vitro without a mesenchymal niche. *Nature.* 2009;459(7244):262-5.
4. Fujii M, Matano M, Toshimitsu K, Takano A, Mikami Y, Nishikori S, et al. Human Intestinal Organoids Maintain Self-Renewal Capacity and Cellular Diversity in Niche-Inspired Culture Condition. *Cell Stem Cell.* 2018;23(6):787-93 e6.
5. Dobin A, Davis CA, Schlesinger F, Drenkow J, Zaleski C, Jha S, et al. STAR: ultrafast universal RNA-seq aligner. *Bioinformatics.* 2013;29(1):15-21.
6. Liao Y, Smyth GK, and Shi W. The R package Rsubread is easier, faster, cheaper and better for alignment and quantification of RNA sequencing reads. *Nucleic Acids Res.* 2019;47(8):e47.
7. Robinson MD, McCarthy DJ, and Smyth GK. edgeR: a Bioconductor package for differential expression analysis of digital gene expression data. *Bioinformatics.* 2010;26(1):139-40.
8. Ge SX, Son EW, and Yao R. iDEP: an integrated web application for differential expression and pathway analysis of RNA-Seq data. *BMC Bioinformatics.* 2018;19(1):534.
9. Li H, and Durbin R. Fast and accurate short read alignment with Burrows-Wheeler transform. *Bioinformatics.* 2009;25(14):1754-60.
10. Zhang Y, Liu T, Meyer CA, Eeckhoute J, Johnson DS, Bernstein BE, et al. Model-based analysis of ChIP-Seq (MACS). *Genome Biol.* 2008;9(9):R137.
11. McLean CY, Bristor D, Hiller M, Clarke SL, Schaar BT, Lowe CB, et al. GREAT improves functional interpretation of cis-regulatory regions. *Nat Biotechnol.* 2010;28(5):495-501.
12. Khan A, and Mathelier A. Intervene: a tool for intersection and visualization of multiple gene or genomic region sets. *BMC Bioinformatics.* 2017;18(1):287.
13. Humphrey SJ, Karayel O, James DE, and Mann M. High-throughput and high-sensitivity phosphoproteomics with the EasyPhos platform. *Nat Protoc.* 2018;13(9):1897-916.
14. Fryer CJ, White JB, and Jones KA. Mastermind Recruits CycC:CDK8 to Phosphorylate the Notch ICD and Coordinate Activation with Turnover. *Molecular Cell.* 2004;16(4):509-20.
15. Haber AL, Biton M, Rogel N, Herbst RH, Shekhar K, Smillie C, et al. A single-cell survey of the small intestinal epithelium. *Nature.* 2017;551(7680):333-9.
16. Elmentaite R, Kumasaka N, Roberts K, Fleming A, Dann E, King HW, et al. Cells of the human intestinal tract mapped across space and time. *Nature.* 2021;597(7875):250-5.
17. Linderman GC, Zhao J, Roulis M, Bielecki P, Flavell RA, Nadler B, et al. Zero-preserving imputation of single-cell RNA-seq data. *Nat Commun.* 2022;13(1):192.

18. Hao Y, Hao S, Andersen-Nissen E, Mauck WM, 3rd, Zheng S, Butler A, et al. Integrated analysis of multimodal single-cell data. *Cell.* 2021;184(13):3573-87 e29.
